# Design Study of a Novel Positron Emission Tomography System for Plant Imaging

**DOI:** 10.3389/fpls.2021.736221

**Published:** 2022-01-18

**Authors:** Emanuele Antonecchia, Markus Bäcker, Daniele Cafolla, Mariachiara Ciardiello, Charlotte Kühl, Giancarlo Pagnani, Jiale Wang, Shuai Wang, Feng Zhou, Nicola D'Ascenzo, Lucio Gialanella, Michele Pisante, Georg Rose, Qingguo Xie

**Affiliations:** ^1^Department of Biomedical Engineering, Huazhong University of Science and Technology, Wuhan, China; ^2^Istituto Neurologico Mediterraneo, NEUROMED I.R.C.C.S, Pozzilli, Italy; ^3^Institute for Medical Engineering and Research Campus STIMULATE, University of Magdeburg, Magdeburg, Germany; ^4^Faculty of Bioscience and Technology for Food, Agriculture and Environment, University of Teramo, Teramo, Italy; ^5^School of Information and Communication Engineering, University of Electronics Science and Technology of China, Chengdu, China; ^6^Yangtze Delta Region Institute of University of Science and Technology of China, Quzhou, China; ^7^Department of Mathematics and Physics, University of Campania L. Vanvitelli, Caserta, Italy; ^8^Department of Electronic Engineering and Information Science, University of Science and Technology of China, Hefei, China

**Keywords:** Positron Emission Tomography, plant stress, functional plant imaging, portable imaging device, plant physiology

## Abstract

Positron Emission Tomography is a non-disruptive and high-sensitive digital imaging technique which allows to measure *in-vivo* and non invasively the changes of metabolic and transport mechanisms in plants. When it comes to the early assessment of stress-induced alterations of plant functions, plant PET has the potential of a major breakthrough. The development of dedicated plant PET systems faces a series of technological and experimental difficulties, which make conventional clinical and preclinical PET systems not fully suitable to agronomy. First, the functional and metabolic mechanisms of plants depend on environmental conditions, which can be controlled during the experiment if the scanner is transported into the growing chamber. Second, plants need to be imaged vertically, thus requiring a proper Field Of View. Third, the transverse Field of View needs to adapt to the different plant shapes, according to the species and the experimental protocols. In this paper, we perform a simulation study, proposing a novel design of dedicated plant PET scanners specifically conceived to address these agronomic issues. We estimate their expected sensitivity, count rate performance and spatial resolution, and we identify these specific features, which need to be investigated when realizing a plant PET scanner. Finally, we propose a novel approach to the measurement and verification of the performance of plant PET systems, including the design of dedicated plant phantoms, in order to provide a standard evaluation procedure for this emerging digital imaging agronomic technology.

## 1. Introduction

*The increasing recurrence of droughts, floods, forest fires, and new pests are a constant reminder that our food system is under threat and must become more sustainable and resilient* (The European Commission, 2020), as stated in the recently approved Green Deal (Sikora, 2021), not only at European level, but on a global scale. It comes as no surprise that the 2020 Nobel Peace Prize has been awarded to the “World Food Program,” thus confirming a series of economic, social, and ecological emergencies concerning food security worldwide. The main objective of the food security program is to increase the production yield of cereals around the globe, ensuring the incremental demand for food, animal feed, and biofuels (Miraglia et al., 2009; Pisante et al., 2012; Prosekov and Ivanova, 2018). Climate change plays here a fundamental role, as the related temperature stress represents the most important factor limiting the production yield of cereals. Stress is in fact generating a complex cascade of severe physiological modifications affecting the exchange of nutrients with the soil and the plant metabolism. The Green Deal rules out fertilizers because of their high pollution potential. Genetic improvement is a necessary but not sufficient part of the strategy (Bailey-Serres et al., 2019). Agronomy is therefore facing one of the most critical challenges of our century.

Indeed, an environmental-friendly engineering system which supports agronomists in (1) understanding the mechanism of temperature stress signaling at functional level, (2) quantitatively and precisely detecting such a mechanism at a very early stage, and (3) intervening before the damage becomes irreversible is far from being achieved (Galieni et al., 2021).

Although in current plant investigations imaging techniques are commonly used for functional analysis, like fluorescence microscopy (Toyota et al., 2018) and Nuclear Magnetic Resonance (Kuchenbrod et al., 1998), they carry important drawbacks in terms of sample disruption during long term observations (Dixit and Cyr, 2003) and poor sensitivity (Chatham and Blackband, 2001). Positron Emission Tomography (PET) proposes a solution to the problem, being a high-sensitive digital imaging technique able to provide a non-invasive 3D visualization of the dynamic flow of nutrients and water within plants (Galieni et al., 2021), that is essential in early stress assessment (Keutgen et al., 2005; Converse et al., 2013; Schmidt et al., 2020; Mincke et al., 2021). Thanks to the easy availability of molecular probes, it has been used to assess changes in the functional mechanisms of plants under different conditions. For instance, recently ^11^C-imaging revealed the spatiotemporal variability of photosynthates translocation into strawberry fruits in response to increasing daylight integrals at leaf surface (Hidaka et al., 2019; Miyoshi et al., 2021). More interestingly, when combined with properly labeled nanoparticles, PET can trace the transport of pollution particles in food with unprecedented precision (Davis et al., 2017). The quantitative measurement of phloem/xylem transport with compartmental modeling remains one of the most striking possibilities of plant PET, and has been shown very promising for the early detection of damages due to climate change (Tsukamoto et al., 2008; Yoshihara et al., 2014; Karve et al., 2015; Partelová et al., 2017; Hubeau et al., 2019a,b; Mincke et al., 2020). The study of fixation and translocation of CO_2_ has also a potential in the identification of novel methods for the improvement of bioproduction in vegetables and fruits (Yamazaki et al., 2015; Kurita et al., 2020).

The key advantage of PET in agronomy, with respect to other imaging techniques, is the possibility of *in-vivo* functional measurements. This feature has also motivated the development of dedicated PET technologies.

**Figure 1:**
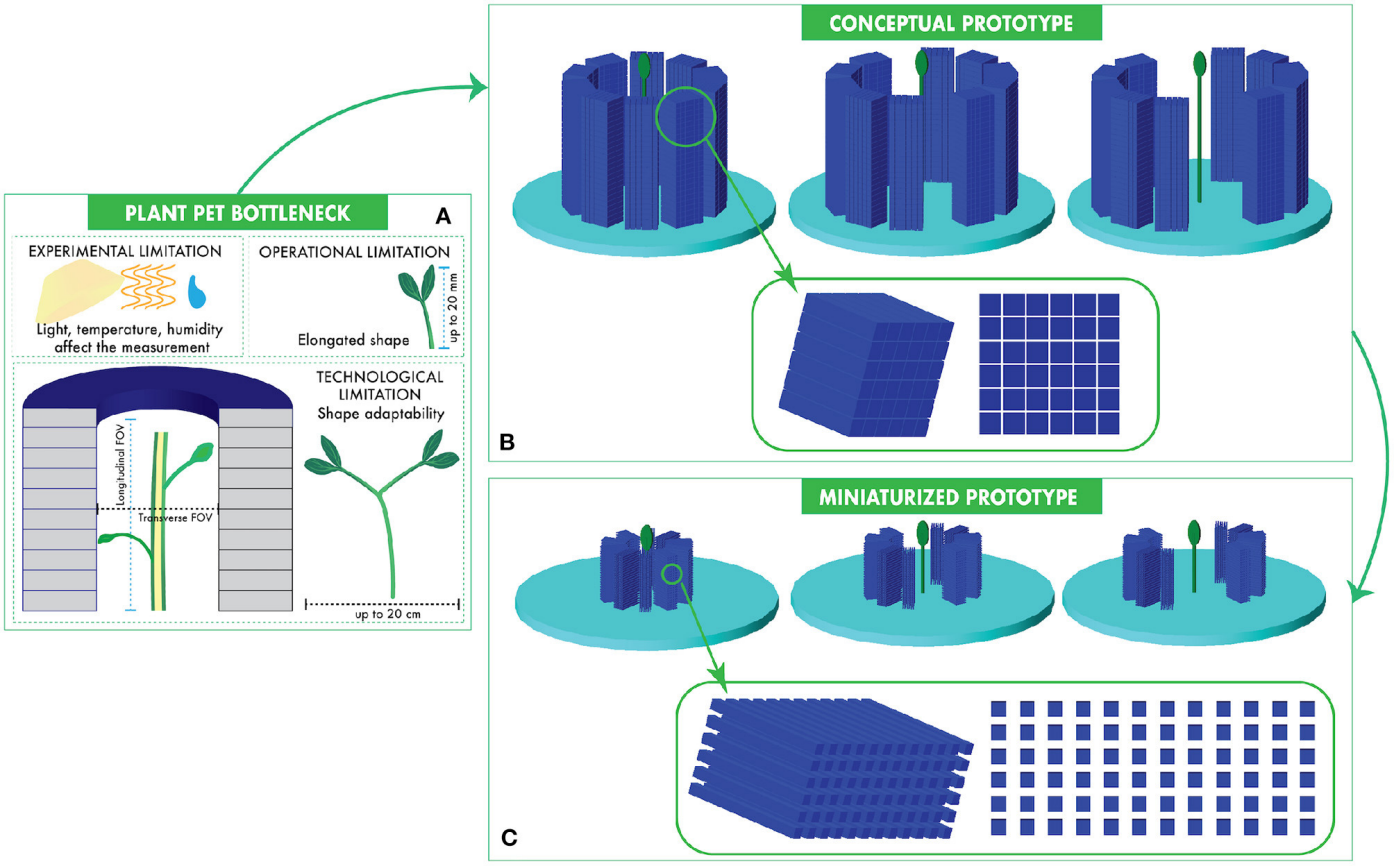
The application of PET to agronomy is limited by experimental, operational, and technological bottlenecks **(A)**. We propose a new concept of a portable plant PET scanner composed of two movable half-cylinders **(B)** and we study its miniaturization options **(C)**. The main parameters of the two systems are summarized in Table 1.

As summarized in Figure 1A, a series of technological limitations are impairing the extension of PET to agronomy and plant science, despite the high potential of this unique functional imaging technique. First, the functional and metabolic mechanisms of plants depend on light exposure, temperature, and humidity, among others. Therefore, the reproducibility of the experiments relies on controllable environmental conditions, which can be obtained only in dedicated growing houses. A plant PET camera needs to be movable to different growing facilities. The electronic and sensing components of a PET system are not seriously affected by the typical heat and humidity conditions of a greenhouse. A conventional air cooling system to avoid overheating of the electronics and a well designed outer shell to avoid excessive humidity exchange with the internal parts of the system are enough to guarantee a proper operation of the machine. However, besides the radiation protection issues, which can be easily overcome with a proper shielding, the complexity of the design of conventional PET systems does not allow transportability. In fact, a typical PET system is an arrangement of multiple sensing modules composed of a detecting unit and a dedicated readout service unit. It means that the bigger is the system, the higher is the number of PET modules needed. The dimension of the system affects also the design and sizing of essential elements like switches, power supplies, clocks, cooling circuits, and shielding. For example, a Raycan Trans-PET® scanner reaches 700 kg in weight and 4.2 m^3^ in volume with its 16,224 crystals (Liang et al., 2020). A dedicated plant PET scanner (1,000–1,500 crystals) may reach size and weight of the sensing and electronic units 15 times smaller. Transportability to greenhouses or growing chambers is pivotal to find the most suitable environment for functional analysis on plants, because we can limit the induced stress on plants by transport and adaptation to conventional PET scanning conditions, minimizing the related measurement bias.

Second, plants need to be imaged in vertical size in order to estimate transport mechanisms in accordance to their physiology. This would imply a vertical extension of the Field Of View (FOV) up to several tens of millimeters without impacting the sensitivity and the spatial resolution performance. Conventional preclinical and clinical systems are developed with a horizontal extension of the FOV due to the supine position of small animals and patients during a PET scan. Several efforts on plant PET investigations have been made so far using conventional scanners (Ariño-Estrada et al., 2019; Ruwanpathirana et al., 2021) but with samples positioned vertically inside the camera: the plant displacement along the radial direction of the FOV causes non-uniformity in terms of spatial resolution. However, there are few attempts of PET scanners with the FOV extended in vertical that are conquering a niche in the field of personalized bioimaging (Norvall et al., 2021; Sakai et al., 2021). That reveals a nice suggestion to move toward the trend of personalized screening approach on plants as well.

Third, the transverse extension of plants depends on the species and on the experimental protocol. While early stress assessment is usually performed with a time-dynamic imaging of small-sized seeds and sprouts approximately 1 week after germination, more complex transport experiments may require larger capability up to few tens of millimeters in diameter. The diameter of the FOV defines the maximal size of the targets and is fixed in conventional PET systems, which are not suitable to an adaptive approach. It is well known that the arabidopsis and plants cultivated for food use like rice, sunflower, and maize have a radial expansion (Dolan et al., 1993; Hochholdinger, 2008; Hochholdinger and Tuberosa, 2009; Mai et al., 2014). At later stages of sprout growth they further develop branches and leaves, that contribute to their radial evolution. Although this feature may justify a PET system with cylindrical geometry, agronomic experiments may necessitate the analysis of multiple plants in a single scan to compare directly samples at the same time. This is particularly important, for instance, in rapeseed and wheat, which get benefits from the tillering and have a growth influenced by the sowing density (Zadoks et al., 1974; Leach et al., 1999; White and Edwards, 2008). Such a requirement justifies a scanner with elongated oval geometry. Since providing multiple scanners with different FOV sizes is a costly solution, shape adaptation plays a key role in plant PET. The final aim is to reach a spatial resolution in any geometrical configuration at the center of the scanner FOV of around 1.8 mm and 1 mm for a conceptual scanner geometry with large crystal cross section and a miniaturized scanner geometry with smaller crystal cross section, respectively.

Besides the application to plant imaging of conventional high resolution digital preclinical PET technologies (Liang et al., 2020), dedicated plant PET scanners addressing these specific issues of agronomy have been proposed. Planar (Kawachi et al., 2006; Streun et al., 2007; Weisenberger et al., 2011), combined cylindrical (Yamaya et al., 2011), and half-cylindrical (Wang et al., 2014) configurations have been recently explored. The development of portable and compact plant PET systems opened up a novel strategy of the geometrical design. For instance, the Open PET system extends the FOV along the vertical axis by displacing two detection rings, including also dedicated Depth Of Interaction (DOI) correction (Yoshida et al., 2012).

The recent advances in compact sensor technologies and fast digital readout strategies (D'Ascenzo et al., 2018, 2020) make it possible to explore even more compact and shape adaptive geometries with full flexibility in response to the needs of agronomy. In this paper, we investigate the expected performance of a novel design of dedicated plant PET systems addressing the specific needs of plant imaging. Furthermore, filling in the existing gap in standard evaluation of clinical systems and agricultural systems, we define here how to adapt the established NEMA standard procedure to the evaluation of plant PET scanners.

The paper is organized as follows: in section 2 we describe the plant PET models, the simulation setup and the analysis methods; in section 3 we show the results of the performance evaluation; in section 4 we discuss the results; in section 5 we report our conclusions.

## 2. Materials and Methods

### 2.1. PET Systems Simulation

#### 2.1.1. Conceptual and Miniaturized Plant PET Geometry

In order to address the above-mentioned issues of PET in agronomy, we propose two possible designs of plant PET systems, which are summarized in Table 1. In the first one, a conceptual prototype (**CONC**), we define the general concept of a plant PET geometry, without overwhelming the technological requirements. A 3D view of the **CONC** prototype is shown in Figure 1B. The required portability and elongation of the system are obtained with a compact design, which extends with a vertical FOV. In order to adapt to the different transverse sizes of plants, the system is composed of two movable half-cylinders with 83.4 mm diameter and 100.8 mm axial length. The system has a vertical axial cylindrical symmetry. The two half-cylinders can be separated at a distance up to 40 mm. The technology requirement of this prototype is conservative. We propose to use PET heads already established for other scanners (D'Ascenzo et al., 2018; Antonecchia et al., 2020). They consist of an array of 6 × 6 Lutetium Yttirium Oxyorthosilicate (LYSO) scintillators. Each crystal has a size of 3.9 × 3.9 × 20mm^3^ and is composed of a mixture of 71.20% Lutetium, 4.01% Yittirium, 6.5% Silicon, 18.1% Oxygen, and 0.19% Cerium. The gap between two contiguous crystals is 0.3 mm in both longitudinal and transverse directions and is filled with BaSO_3_ to reduce the light cross-talk between adjacent crystals. Each layer of the half-cylinder composed of five heads. Four head-layers are displaced vertically. Therefore, each half-cylinder counts 24 crystal layers formed by 30 scintillators. A stack of scintillators laying on the same horizontal plane of the camera is called *ring*. Therefore, the scanner counts 24 rings formed by 60 scintillators (30 crystals in each half-cylinder). The entire scanner is composed of 1, 440 crystals.

**Table 1:** Geometrical features of the conceptual and miniaturized systems.

	**CONC**	**MINI-13**	**MINI-16**	**MINI-20**
Crystal cross-section [mm]	3.9	1.0	1.0	1.0
Crystal length [mm]	20.0	13.0	16.0	20.0
Transverse crystal pitch [mm]	4.2	1.6	1.6	1.6
Longitudinal crystal pitch [mm]	4.2	1.9	1.9	1.9
Scanner diameter [mm]	83.4	30.5	30.5	30.5
Scanner axial length [mm]	100.8	45.6	45.6	45.6
Number of scanner rings	24	24	24	24
Number of crystals per ring	60	60	60	60

The second prototype (**MINI**) further explores the portability potential of plant PET, by proposing a miniaturization of the **CONC** system (Figure 1C). The key component of the **MINI** prototype is the PET head composed of crystals with tiny cross section, as low as 1 × 1 mm^2^. The crystal length is considered an optimization parameter. We studied three configurations, namely 13 mm (**MINI-13**), 16 mm (**MINI-16**), and 20 mm (**MINI-20**) long scintillators. In practice, a 1:1 readout of these small crystals by using modern silicon detectors, such as Silicon Photomultipliers (SiPM), requires a careful consideration of the packaging options of SiPM arrays. Following a realistic engineering design, the gap between neighboring scintillator is 0.6 mm in the transverse direction and 0.9 mm in the longitudinal direction. The **MINI** system is composed of two half-cylinders with an axial length of 45.6 mm and a diameter of 30.5 mm. Like the **CONC** system, it counts a total of 1,440 scintillators grouped in 24 detecting rings with 60 crystals per ring (30 crystals in each half-cylinder).

**Figure 2:**
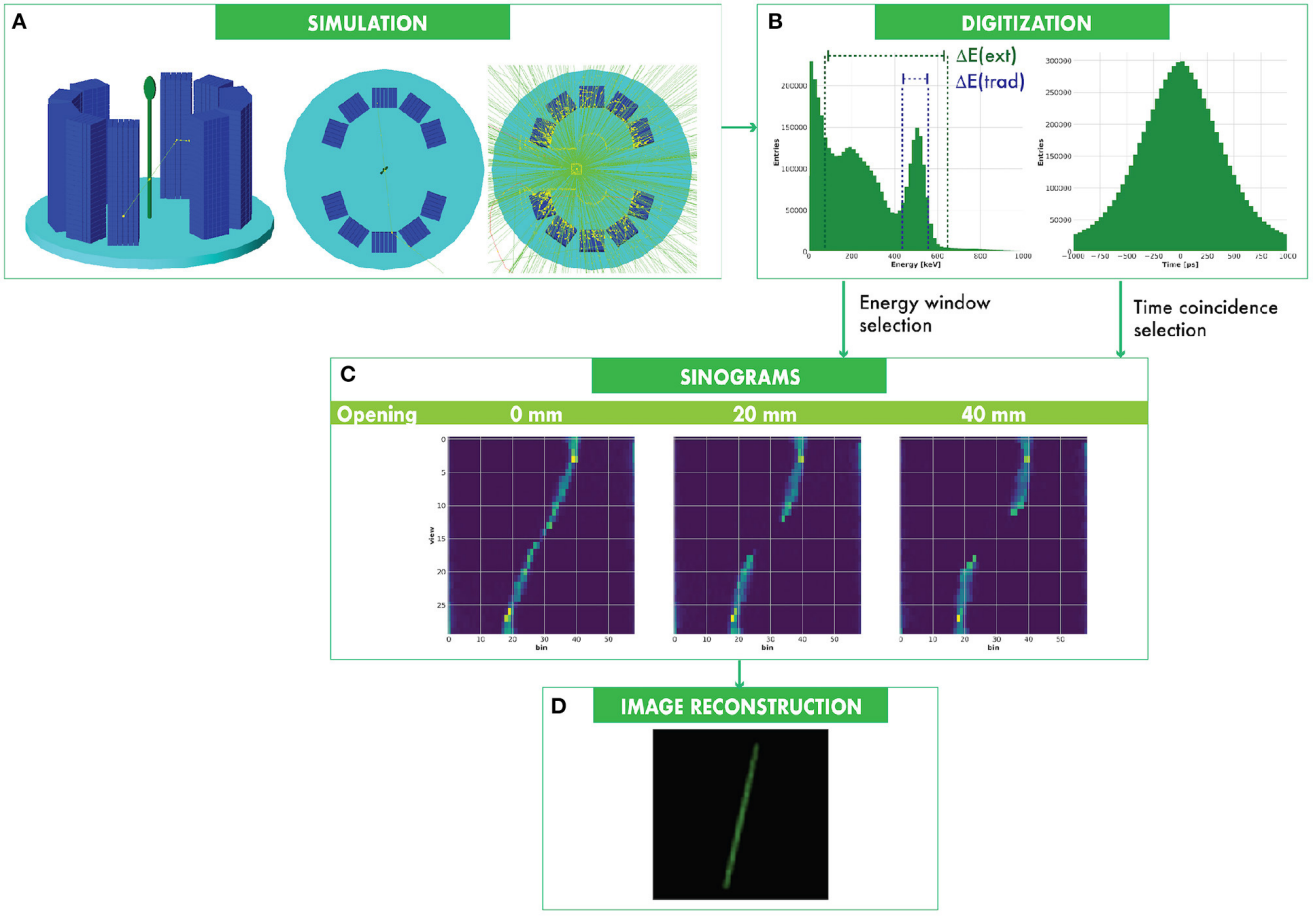
Plant PET simulation and data processing. A plant phantom is placed inside the FOV. A β^+^ emitter in the phantom is simulated. Two γ-rays are generated from the positron annihilation and are detected in the PET sensors **(A)**. The energy deposited in each PET sensor and the detection time are used to discriminate coincident events **(B)**, which are ordered in the sinograms **(C)**. 3D images of the distribution of the radioactive source in the phantoms are reconstructed from the sinograms **(D)**.

We modeled the systems in the GEANT4 simulation framework (Agostinelli et al., 2003; Allison et al., 2016). The simulation includes the radioactive decay of the unstable β^+^ emitters and the electromagnetic physics characterizing the positron annihilation and the further interaction mechanisms of the two annihilation 511 keV γ-rays in the scintillators (Figure 2A). The radioactive decay of the fraction of ^176^Lu in LYSO is not considered in the simulation since the intrinsic radioactivity poorly affects the evaluations of camera performances, mainly due to the small number of crystals we used for the scanner architecture. In fact, the count rate derived from intrinsic radioactivity in a ^176^Lu-based crystal is approximately 300Bq/ml (Enr-quez-Mier-y Terán et al., 2020). Considering the total crystal volume for **CONC** system (438 ml) and for **MINI** systems (from 18.7 ml to 28.8 ml), the total intrinsic radioactivity can be estimated at most 8 kBq for **MINI** systems and 131 kBq for **CONC** system. Since testing radioactivities are >1MBq, the intrinsic radioactivity can be considered negligible for performance simulations. The generation of scintillation photons in the crystals and the further propagation and detection are not included in the simulation. The emission energy of the β^+^ is of critical importance in plant PET, as the plant tissues are thinner than the average range of positrons. In order to perform a precise calculation, we used the Evaluated Nuclear Structure Data File (ENSDF) libraries (Burrows, 1990) for the proper simulation of the decay of the radio-nuclei.

#### 2.1.2. Data Processing and Image Reconstruction

The simulation outputs the deposited energy, the detection time, and the crystal position of each detected γ-ray. As mentioned above, the propagation of scintillation optical photons and their detection in a realistic photosensor is not included in the simulation. In order to take into account a realistic environment and following previous observations in similar systems (D'Ascenzo et al., 2020), we smear the value of the deposited energy and of the detection time with a Gaussian with 15% resolution (FWHM) and 350 ps (FWHM), respectively. The simulated energy distribution deposited by the γ-ray in the crystals is shown in Figure 2B.

Annihilation γ-rays may undergo Compton scattering and deflect their trajectory before detection, causing a bias in the determination of the positron emission point and image blurring. In order to disregard Compton scattered events hitting the crystals, only events with a deposited energy laying within an energy window Δ*E* are selected. We consider here two different approaches to the determination of the energy window. On the one hand, we define a conservative and traditional energy window centered at the 511 keV photoelectric peak Δ*E*(*trad*) = (350, 650) keV. On the other hand, we consider that the probability of Compton scattering in the soft and thin plant tissues is very low. Therefore, we study also the possibility of an extended energy window Δ*E*(*ext*) = (50, 750) keV, which would be beneficial in order to increase the overall efficiency of the technique. We chose the lower energy cut at 50 keV because it guarantees a signal from 40 to 80 photoelectrons at least, high enough to noticeably exceed the electronic noise due to dark current of detectors that is not higher than 7.5 photoelectrons (D'Ascenzo and Saveliev, 2011). We define the energy-selected events as *singles*. We consider two singles in coincidence if they occur within a time window of 2 ns. We include in the simulation a paralyzable crystal dead time of 200 ns and a paralyzable system global dead time of 20 ns.

A coincidence pair between two crystals identifies a unique 3-dimensional line of response (LOR), along which the positron emission took place. LORs are organized in 24 × 24 sinograms, each of which is a 2D matrix of pixels representing all the possible LORs of a specific transverse slice of the camera including the oblique slices described by different rings. The position of one LOR in a sinogram is uniquely identified by a couple of coordinates called *bins* and *views*, that represent the information about the radial distance of the LOR from the FOV center and the angular displacement of the LOR, respectively. One sinogram is 2-dimensional containing (*N*−1) × (*N*/2) pixels, where *N* = 60 is the number of crystals per ring, (*N*−1) is the number of *bins*, while (*N*/2) is the number of *views*. An example of sinogram measured at different separations of the two half-cylinders is shown in Figure 2C.

For the reconstruction of PET images we computed the System Response Matrix (SRM) based on a high resolution Bayesian method for 3D Maximum *a posteriori* (MAP) image reconstruction (Qi et al., 1998). We noticed that the Filtered Back Projection algorithm (FBP) suffers the geometrical singularities of our scanners in opened configurations: because of the missing solid angles between the two camera halves, it returns artifacts and distortions in image reconstruction. For such reason, we opted for reconstructing the images from the sinograms by using a 3D ordered subset expectation minimization algorithm (3D-OSEM), that is less sensitive to the missing angles problem. For completeness, we listed also the FBP results in the Supplementary Materials. The voxel size of the reconstructed image is 0.5 × 0.5 × 0.5 mm^3^. The reconstructed image of the plant phantom is shown in Figure 2D.

### 2.2. NEMA Standards Characterization

The National Electrical Manufacturers Association (NEMA) defines quantitative standards only for the evaluation of preclinical (NEMA, 2008) and clinical scanners (NEMA, 2007). Currently there is no certified standard protocol for testing the performances of tomographs that are made for plant screening purpose. Moreover, beside the target they are built for, the classification of PET scanners is based on the dimensions of the machine. For instance, a PET device for small-animals must have a transverse FOV at least 33.5 mm large according to NEMA. Though the **CONC** system may fit such constraint, the **MINI** systems are out of that scale. Lastly, current standards provide only human or animal shaped phantoms that are not dimensionally suitable for regular plant simulation. Therefore, for our tests on plant PET systems, we took inspiration from NEMA for small-animal tomographs and adjusted the current protocols when needed for more meaningful measurements of such new typology of PET cameras. We claim the necessity to define a novel standard procedure for the evaluation of plant PET systems, which is closer to the experimental needs of agronomy.

#### 2.2.1. Sensitivity

**Figure 3:**
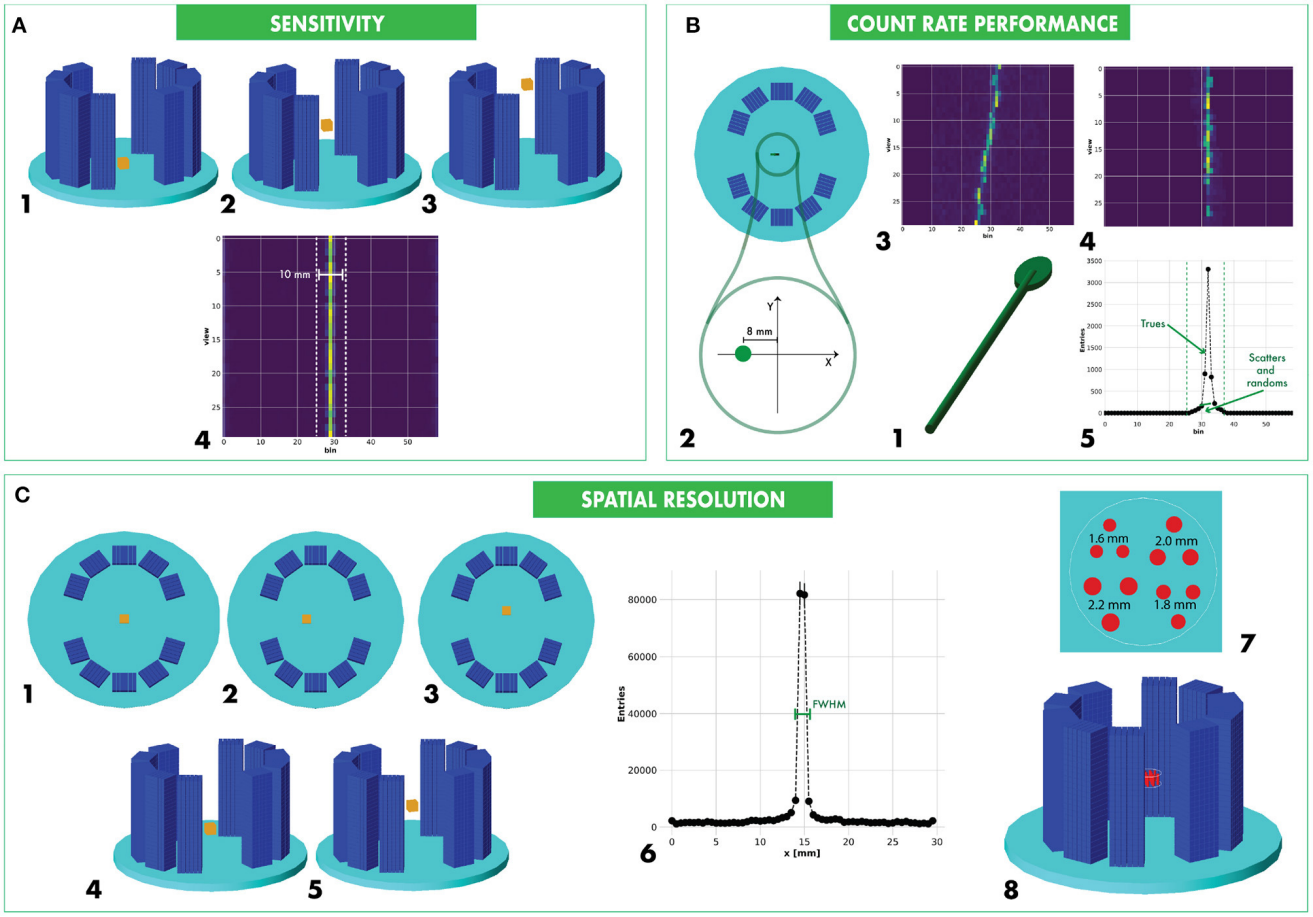
Modified NEMA standards for Plant PET characterization: measurement of the sensitivity **(A)**, count rate performance **(B)**, and spatial resolution **(C)**.

The sensitivity of a PET camera defines the fraction of positron annihilation events detected as true coincidence events in response to the activity in the FOV. The method, radionuclide setting and source distribution come directly from the standard method (NEMA, 2008). For data collection, calculation and analysis we adopted the procedures described in Antonecchia et al. (2020) for sensitivity measurements from simulated data. We simulated a point-like ^22^Na source with a 0.3 mm radius positioned at the center of a PMMA cube with 10 mm side length. As shown in Figures 3A-1, 3, we moved the radioactive source along the longitudinal axis by 4 mm and 2 mm steps in the **CONC** and **MINI** system, respectively. We simulated 2.5·10^6^ events for each position.

**Figure 4:**
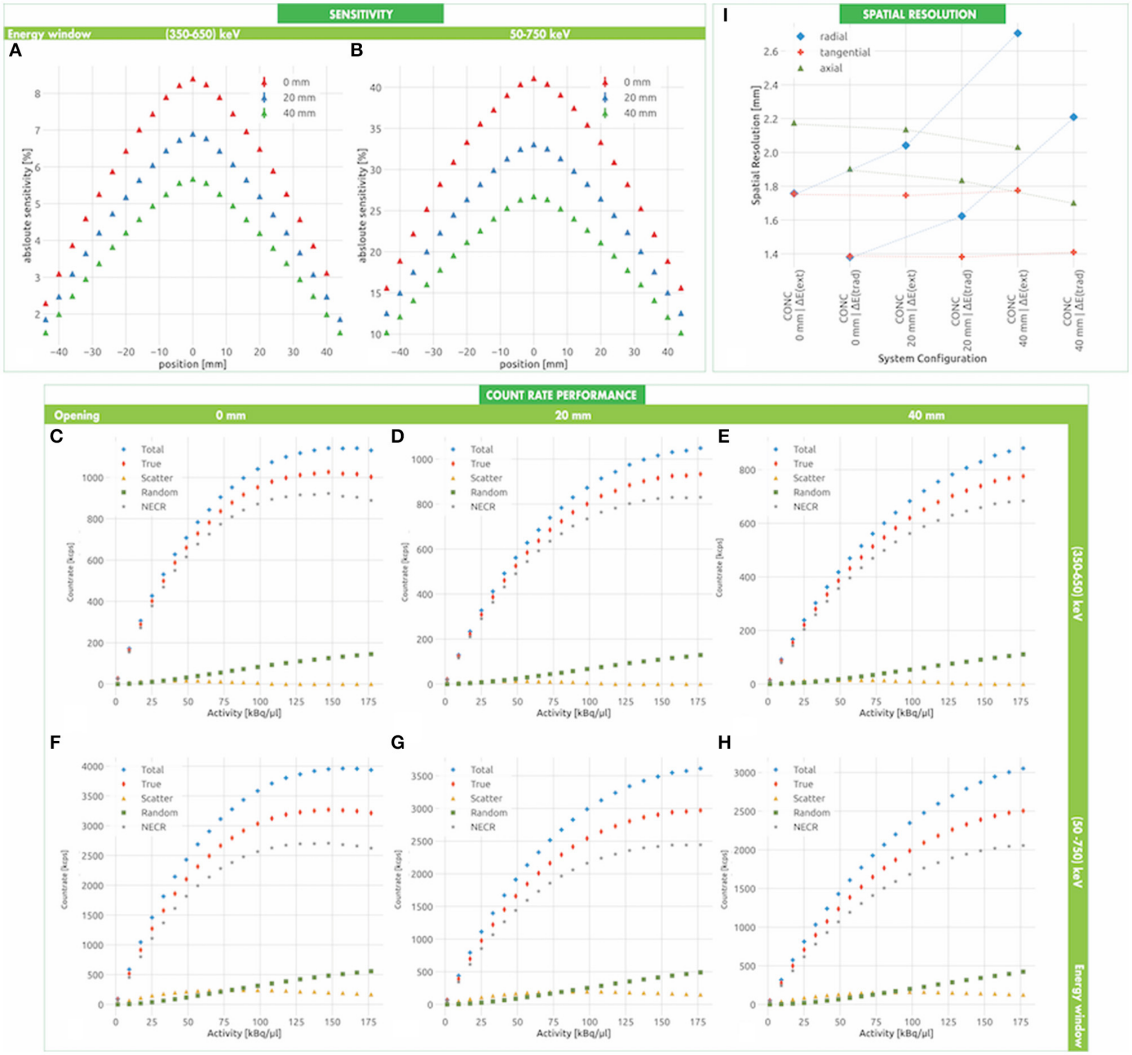
Conceptual system expected performances. The sensitivity is estimated for the conventional **(A)** and the enlarged **(B)** energy window at three openings of the two half-cylinders. Similarly, the count rate figure is estimated at three openings of the two half-cylinders for the conventional **(C–E)** and enlarged **(F–H)** energy window, respectively (see also Supplementary Tables 2, 3). The radial, tangential and longitudinal spatial resolution at the center of the FOV are reported in **(I)** for different energy windows and opening angles (see also Supplementary Tables 4–7). The dashed lines represent the spatial resolution profiles related to the same energetic window over different openings.

The analysis was performed on all the 2D sinograms measured at the *nth* source position. An example of the sinogram is shown in Figure 3A-4. We identified the hottest *bin* for each *view* and we retrieved the total number of counts *C*_*n, out*_ in the group of *bins* within a 10 mm window (Figure 3A-4). The sensitivity is defined as:


(1) $$\begin{array}{l} S_{n}\pm \Delta S_{n}=\frac{C_{n,out}}{C_{in}\times Na_{br}}\pm \frac{\sqrt{C_{n,out}}}{C_{in}\times Na_{br}}\\ =\frac{C_{n,out}}{2.5\times 10^{6}\times 0.906}\pm \frac{\sqrt{C_{n,out}}}{2.5\times 10^{6}\times 0.906} \end{array}$$

where *C*_*in*_ is the total number of events we simulated for each phantom position, *Na*_*br*_ is the β^+^ decay branching ratio of the ^22^Na isotope and the statistical error takes in consideration only the Poisson fluctuation.

#### 2.2.2. Count Rate Performance

The count rate measurement of a PET scanner indicates the ability of the machine to acquire events especially at high activity when the acquisition limitations, due to the dead time losses, become significant. Moreover, this calibration includes also the computation of the Compton scattered and random coincidence event rates, which disturb the quality of the reconstructed image.

Following NEMA (NEMA, 2008), we measure the count rate of the scanner in response to a line-shaped source, normally [^18^F]-FDG, inserted in the FOV. Key to the measurement is a phantom, in which the radioactive tracer is embedded. Its purpose is to emulate the density and size of a biological system. It allows to have a realistic approximation of the Compton scatter probability. Existing NEMA standards define cylindrical PMMA phantoms emulating mice, rats, monkeys, and human subjects, which are significantly larger than plants. Our goal is to introduce a new standard that provides a feasible plant-like structure.

As shown in Figure 3B-1, we propose a phantom model representative of a dicotyledon plant, with a shape that is inspired by zucchini sprouts. It is composed of a mixture of water and cellulose as the main constituents, in the following proportions: 80% H_2_O (Lumen, 2021) and 20% C_6_H_12_O_5_, with a density of 1.2g/cm^3^. As shown in Figure 3B-1, the phantom has a solid structure with a cylindrical basis (the stem) and an elliptical disk (the leaf). The stem length should cover the entire longitudinal FOV of the system and is 100 mm and 40 mm for the evaluation of **CONC** and **MINI** system, respectively. The stem radius is 1.5 mm. The leaf is 20 mm long, 10 mm large, and 2 mm thick. A capillary tube with a radius of 0.25 mm is filled with the tracer. Compared to the capillary lumen, we fixed the stem with a diameter 6 times bigger and the leaf with a thickness 4 times bigger. We imposed constraints on the capillary dimensions, based on small syringe diameters used for tracer injection and the average resolution of device manufacturing.

We placed the phantom along the scanner parallel to the longitudinal direction, with the geometrical midpoint crossing the axial center of the FOV and with a radial offset of 8 mm and 3 mm for the **CONC** and **MINI** system, respectively (Figure 3B-2). The leaf is therefore positioned along the longitudinal axis with the center placed with a 50 mm and 20 mm offset from the FOV center for the **CONC** and **MINI** systems, respectively.

We randomly generated ^18^F atoms into the capillary volume, with an initial activity of 150.0 MBq. We simulated 21 independent 0.5 s wide time frames spanning the decay of the radio-nuclei down to an activity of 1.2 MBq. The simulated number of events for each time frame is reported in the Supplementary Table 1.

**Figure 5:**
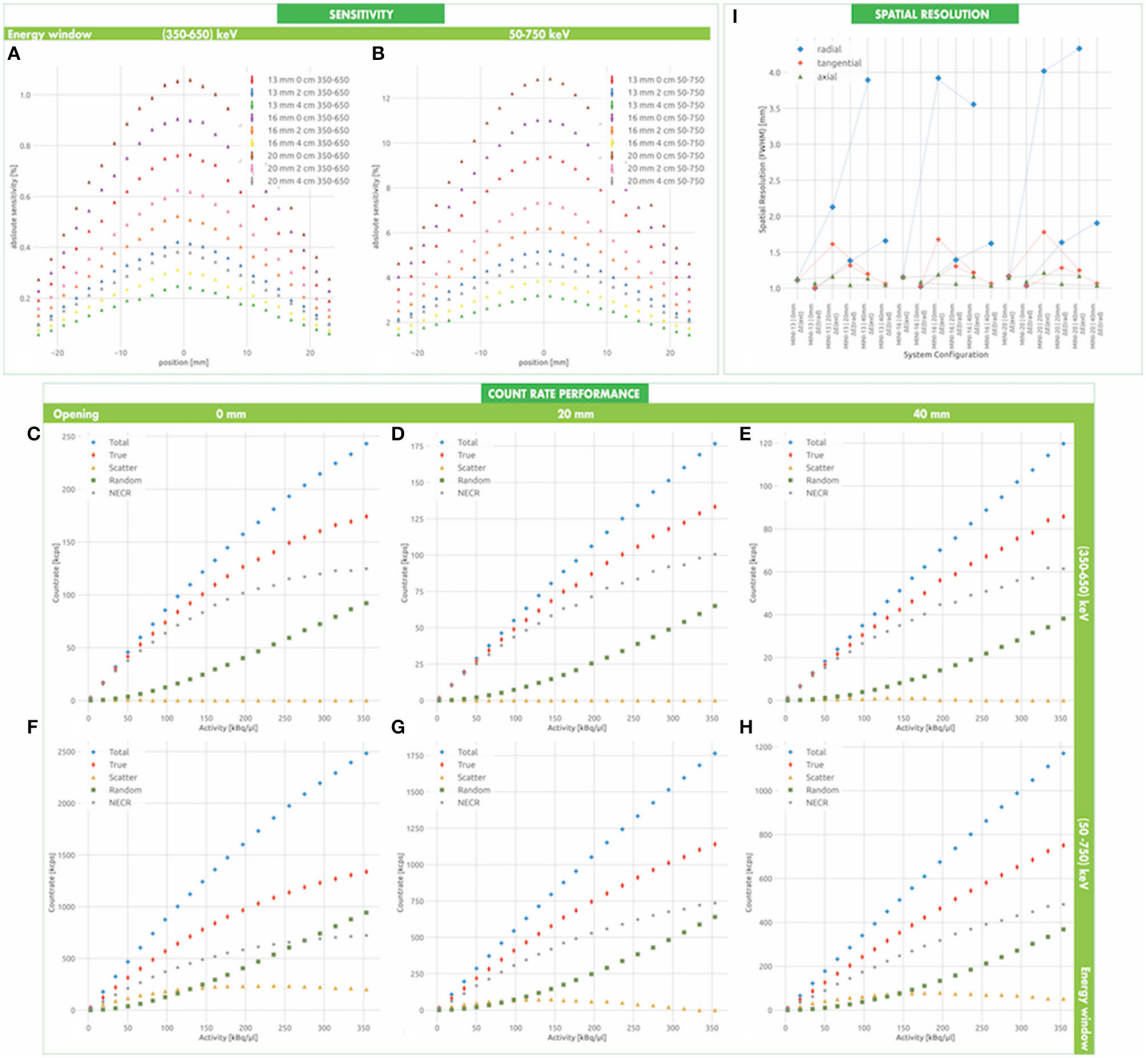
Miniaturized system expected performances. The sensitivity is estimated for the conventional **(A)** and the enlarged **(B)** energy window at three openings of the two half-cylinders. Similarly, the count rate figure is estimated at three openings of the two half-cylinders for the conventional **(C–E)** and enlarged **(F–H)** energy window, respectively (see also Supplementary Tables 8, 9). The radial, tangential and longitudinal spatial resolution at the center of the FOV are reported in **(I)** for different energy windows and opening angles (see also Supplementary Tables 10–13). The dashed lines represent the spatial resolution profiles related to the same energetic window over different openings.

We performed the analysis on the collected sinograms for each time frame, adapting the NEMA procedure to the dimensions of **CONC** and **MINI** scanners. As shown in Figure 3B-3, for each pair of rings the sinogram is a sinusoidal line. A zero-suppression was first done by selecting a window of width of 11 *bins* and 13 *bins* across the center of the FOV, for the **CONC** and **MINI** system, respectively. The *bin* windows are different because the pixel dimension is larger for the **CONC** system, due to the camera diameter that is wider than the diameter of the **MINI** system. For each *view*, we spotted the pixel having the highest value to determine the center of the line source response. We shifted all the pixels of each *view* in order to align all the pixels containing the maximum values at same central *bin* (Figure 3B-4). Finally, we created the sum projection array, where each value corresponds to the sum of pixels placed at the same *bin* (Figure 3B-5):


(2) $$\begin{array}{l} C\left(bin_{i}\right)\pm \Delta C\left(bin_{i}\right)=\overset{N_{views}}{\underset{\nu =1}{\sum }}C\left(bin_{i}-bin_{max}\left(\nu \right),\nu \right)\\ \pm \overset{N_{views}}{\underset{\nu =1}{\sum }}\sqrt{C\left(bin_{i}-bin_{max}\left(\nu \right),\nu \right)} \end{array}$$

where *bin*_*i*_ is the pixel correspondent to a specific *i-bin* and *bin*_*max*_(ν) is the reference *bin* correspondent to the position of the pixel with the greatest value in the *view*=ν. The statistical error considers only the Poisson fluctuations in each pixel of the sinogram. We analyzed this resulting histogram, by selecting a window of 4 *bins* for **CONC** system and 6 *bins* width for **MINI** system. At the edges of the window we individuated two pixels with values *C*_*R*_ and *C*_*L*_ and a linear interpolation of these two points defined two areas in the sum projection plot: the upper and lower areas estimate, for each *jth* sinogram, the number of true and noise events, respectively. The value of true events is $C_{j}^{true}\pm \Delta C_{j}^{true}$, while the number of noise events is $C_{j}^{scat+rnd}\pm \Delta C_{j}^{scat+rnd}$, where $\Delta C_{j}^{true}=\sqrt{C_{j}^{true}}$ and $\Delta C_{j}^{scat+rnd}=\sqrt{C_{j}^{scat+rnd}}$. The noise events include random and scatter coincidences. The total number of counts in the plot represents the total number of coincident events $C_{j}^{TOT}$ for the *jth* sinogram, and acquisition *k*:


(3) $$\begin{array}{l} C_{j,k}^{TOT}\pm \Delta C_{j,k}^{TOT}=\overset{N_{bins}}{\underset{i=1}{\sum }}C_{j,k}\left(bin_{i}\right)\pm \overset{N_{bins}}{\underset{i=1}{\sum }}\Delta C_{j,k}\left(bin_{i}\right) \end{array}$$

where $\Delta C_{j,k}\left(bin_{i}\right)=\sqrt{C_{j,k}\left(bin_{i}\right)}$. Addressing the total number of sinograms *N*_*S*_ = 24 ×24 = 576, for each time frame acquisition *k* we calculated the total $R_{k}^{TOT}$ and true $R_{k}^{true}$ count rates as follows:


(4) $$\begin{array}{l} R_{k}^{TOT}\pm \Delta R_{k}^{TOT}=\overset{N_{S}}{\underset{j=1}{\sum }}\frac{C_{j,k}^{TOT}}{T_{frame}}\pm \overset{N_{S}}{\underset{j=1}{\sum }}\frac{\Delta C_{j,k}^{TOT}}{T_{frame}} \end{array}$$

(5) $$\begin{array}{l} R_{k}^{true}\pm \Delta R_{k}^{true}=\overset{N_{S}}{\underset{j=1}{\sum }}\frac{C_{j,k}^{true}}{T_{frame}}\pm \overset{N_{S}}{\underset{j=1}{\sum }}\frac{\Delta C_{j,k}^{true}}{T_{frame}} \end{array}$$

When the activity is lower than a few MBq, the probability of random coincidences is negligible and the noise events are determined only by Compton scattering. With this approximation, the scatter fraction (SF), which defines the ratio between scattered and total coincidences, is:


(6) $$\begin{array}{l} SF\pm \Delta SF=\overset{N_{S}}{\underset{j=1}{\sum }}\overset{21}{\underset{k=19}{\sum }}\frac{C_{j,k}^{scat}}{C_{j,k}^{scat}+C_{j,k}^{true}}\\ \pm \overset{N_{S}}{\underset{j=1}{\sum }}\overset{21}{\underset{k=19}{\sum }}\frac{C_{j,k}^{true}\Delta C_{j,k}^{scat}+C_{j,k}^{scat}\Delta C_{j,k}^{true}}{{\left(C_{j,k}^{true}+C_{j,k}^{scat}\right)}^{2}} \end{array}$$

Finally, the random event rate is calculated according to the methodology suggested by NEMA, when scanners do not directly measure random coincidences:


(7) $$\begin{array}{l} R_{k}^{rnd}\pm \Delta R_{k}^{rnd}=\overset{N_{S}}{\underset{j=1}{\sum }}\frac{C_{j,k}^{TOT}-\frac{C_{j,k}^{true}}{1-SF_{j}}}{T_{frame}}\\ \pm \overset{N_{S}}{\underset{j=1}{\sum }}\frac{\Delta C_{j,k}^{TOT}+\frac{\Delta C_{j,k}^{true}}{1-SF_{j}}+\frac{C_{j,k}^{true}\Delta SF_{j}}{{\left(1-SF_{j}\right)}^{2}}}{T_{frame}} \end{array}$$

The Noise Equivalent Count Rate (NECR) quantifies the signal strength vs. the Compton and scatter background. We estimated it as:


(8) $$\begin{array}{l} NECR_{k}\pm \Delta NECR_{k}=\overset{N_{S}}{\underset{j=1}{\sum }}\frac{{\left(C_{j,k}^{true}\right)}^{2}}{C_{j,k}^{TOT}\times T_{frame}}\\ \pm \overset{N_{S}}{\underset{j=1}{\sum }}\frac{\left(2C_{j,k}^{true}C_{j,k}^{TOT}\right)\Delta C_{j,k}^{true}+{\left(C_{j,k}^{true}\right)}^{2}\Delta C_{j,k}^{TOT}}{{\left(C_{j,k}^{TOT}\right)}^{2}\times T_{frame}} \end{array}$$

#### 2.2.3. Spatial Resolution

The spatial resolution of a PET camera is the ability for the system to distinguish two points of the reconstructed image. Using a compact source we could measure the widths of the point response functions from the reconstructed image. The method for the quantification of the spatial resolution is described in Figure 3C. We used the same phantom as we did for the sensitivity measurement but, differently from current standards, we decided to distribute the source in a more extended spatial range. In fact, as the two half-cylinders composing the plant PET scanners can be displaced, the rotational symmetry of conventional cylindrical PET geometries cannot be applied and the existing NEMA standard (NEMA, 2008) should be adapted to this new situation. We propose to move the phantom at 6 radial positions along the two transverse directions in the X-Y plane (Figures 3C-1, 3), both at the center (Figure 3C-4) and at 1/4 of the longitudinal FOV (Figure 3C-5). While the X-Y measurement will be sensitive to the artifacts introduced by the asymmetry of the detector, the longitudinal displacement will enhance any not-uniformity of the response in the FOV. We simulated 2.5·10^6^ events per phantom position. The collected singles were sorted and processed for coincidence detection and sinogram construction.

**Figure 6:**
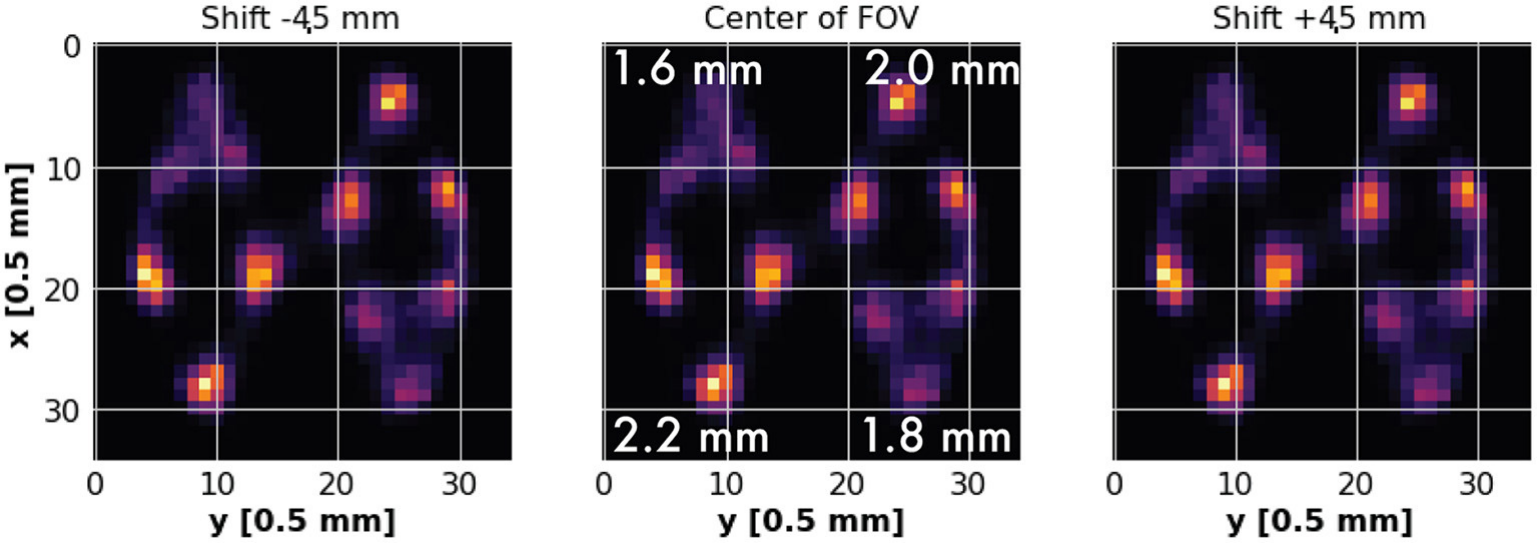
De Renzo imaging of the CONC system. A De Renzo phantom is placed at the center of the FOV and at a shift of ±4.5 mm along the transverse axis of the system.

We analyzed the reconstructed images of the source placed at each spatial point. As shown in Figures 3C-6, we projected the reconstructed image on the two transverse axes and on the longitudinal axis of the FOV. For each of the 3 projections, we determined the greatest value and we involved the two closest points in order to describe a parabolic fitting function. We determined the peak value of the fitting function and the two points of the function correspondent to the half of the peak value *C*_*l*_ and *C*_*r*_, then we calculated the FWHM as the distance in millimeters of the two points above *D*(*C*_*l*_, *C*_*r*_). The estimation is affected by the statistical Poisson error, which we compose according to conventional propagation of errors methods.

### 2.3. De Renzo Imaging

**Figure 7:**
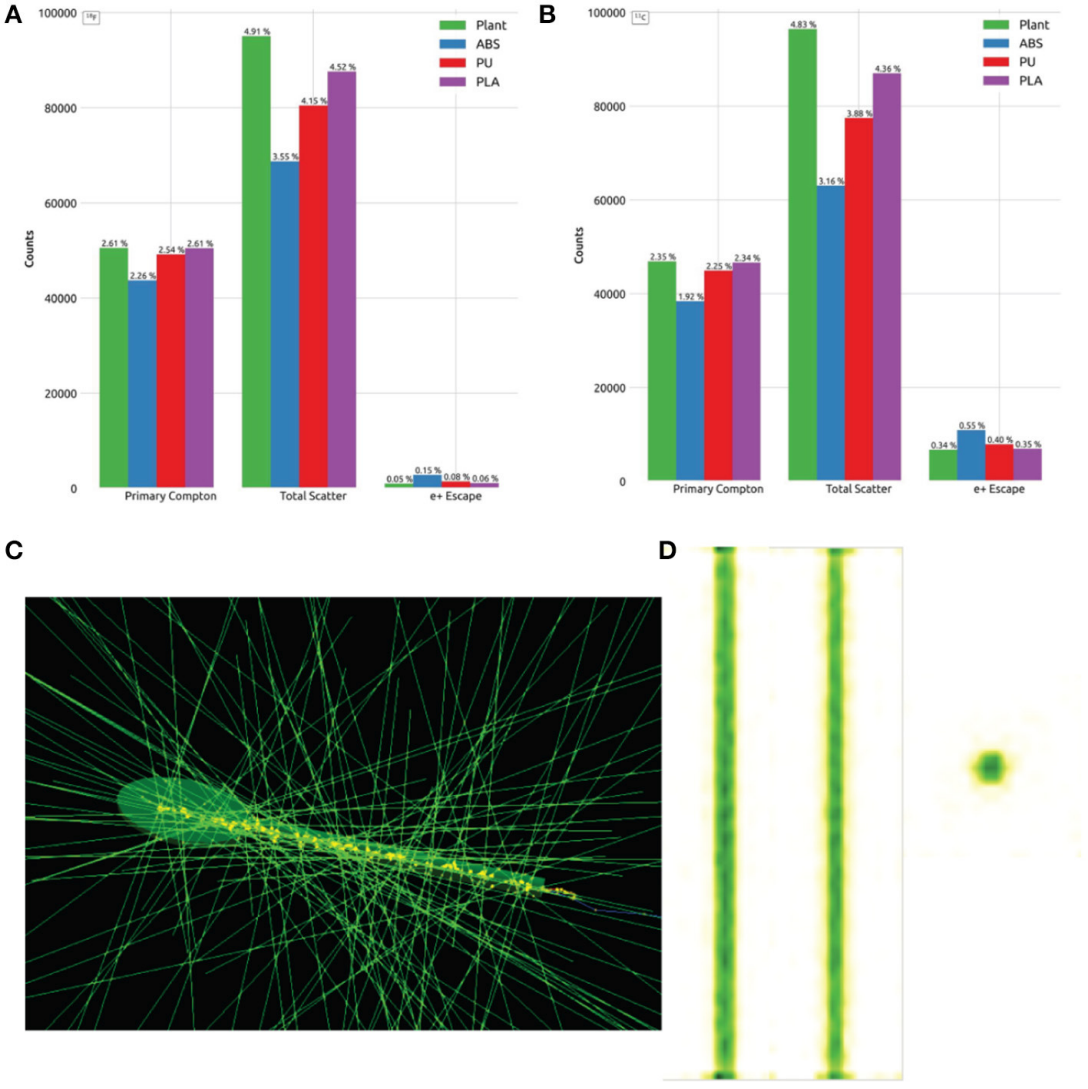
Plant phantom imaging. Distribution of the Scattering and positron escape events using ^18^F **(A)** and ^11^C **(B)**, a simulation frame **(C)** and a typical PET image of the stem of the plant phantom **(D)**. A movie showing the reconstructed image at different angles is found in the Supplementary Material.

We performed a further analysis on spatial resolution through the simulation and image reconstruction of a De Renzo phantom. The phantom we designed is a PMMA disk with a 18 mm diameter and 4 mm height, exhibiting different hole patterns with varying diameters and spacing. The phantom features 4 different hole sizes arranged in triangles around the center of it, while holes within groups are spaced exactly one diameter apart. The diameters of each group of holes are 1.6 mm, 1.8 mm, 2.0 mm, 2.2 mm, while the holes depth is 4 mm for all. We arranged 3 holes with the same diameter for each group. A representation of the De Renzo we designed is shown in Figure 3C-7. Among all the possible configurations, we simulated the phantom in the **CONC** system when the two hemicylinders are separated by a 20 mm gap. We initialized events to simulate in each well according to the volume of the related well, in order to normalize the volumetric radioactivity. The number of simulated events per well are summarized in Table 2. We took data displacing the phantom for 3 different positions inside the FOV: one acquisition at center of the FOV and two acquisitions moving the De Renzo along the radial axis by 4.5 mm offset on both directions across the center of the FOV. Moving the phantom of just 4.5 mm on both direction of the radial axis guarantees the displacement of the holes between 4.5 mm and 13.5 mm far from the FOV center, enabling the evaluation of the spatial resolution in a range from 18 mm to 27 mm across the FOV center. The setup is shown in Figure 3C-8. The selection of *singles* was done using the Δ*E*(*ext*) window.

**Table 2:** De Renzo phantom simulation features.

**Group localization**	**Hole diameter [mm]**	**Volume [*mm*^3^]**	**Holes per group**	**Simulated events**
Top-Left	1.6	8.04	3	5289257
Bottom-Right	1.8	10.18	3	6694215
Top-Right	2.0	12.57	3	8264462
Bottom-Left	2.2	15.21	3	10000000

### 2.4. Plant Phantom Imaging

The characterization of the plant PET system relies on a proper plant phantom. In order to verify the feasibility of the novel proposed phantom, we considered different materials, typically used in biomedical engineering: Acrylonitrile butadiene styrene (ABS), Polyurethane (PU), and Polylactic Acid (PLA). ^18^F and ^11^C are the typical radionuclides used to label tracers for plant studies. The maximal energy of the emitted positrons is approximately 0.634 MeV and 0.960 MeV, respectively. Therefore, we expect different maximal positron ranges of 2.4 mm and 4.2 mm, respectively (Teuho et al., 2020). We studied the effects of scattering and positron escape in the different materials. In one simulation, we initialized 1 million ions of ^18^F inside the capillary of the phantom and tracked the cross sections of the γ-rays generated by the positrons annihilations. In a second simulation we initialized 1 million ions of ^11^C within the same setup. We evaluated both the number of 511 keVγ-rays having at least one Compton scattering and the total amount of all photoelectric and Compton scattering inside the phantom (labeled as *Primary Compton* and *Total Scattering*, respectively). One more feature we evaluated is the positron escape, a common phenomenon involving bodies whose thickness is shorter than the radiotracer mean range (Alexoff et al., 2011). Since the plant is confined in a volume whose size is comparable with the positron range of both radiotracers, we wanted to estimate the number of generated positrons that is likely to escape outside the injected volume without annihilating in the designed plant phantom.

## 3. Results

### 3.1. Conceptual System

#### 3.1.1. Sensitivity

The estimated sensitivity of the conceptual system is shown in Figure 4A for the conventional energy window Δ*E*(*trad*) and in Figure 4B for the extended energy window Δ*E*(*ext*). Its value reaches a maximum at the center of the FOV and decreases linearly following the typical expectations in systems with translational axial symmetry. When the half-cylinders are closed and the energy window Δ*E*(*trad*) is used, the peak and mean sensitivity are (8.400 ± 0.002)% and (5.760 ± 0.002)%, respectively. When the half-cylinders are displaced from each other, the reduced solid angle coverage causes up to 35% decrease of the sensitivity. The extension of the energy window is beneficial. We observe that, if the half-cylinders are closed and the energy window Δ*E*(*ext*) is used, the peak and mean sensitivity are (41.091 ± 0.004) %and (30.200 ± 0.004)%, respectively, thus exhibiting a almost 5-fold gain with respect to Δ*E*(*trad*).

#### 3.1.2. Count Rate Performance

The count rate performance of the conceptual system in a conventional configuration, with closed half-cylinders and small energy window Δ*E*(*trad*) is shown in Figures 4C–E. The system exhibits a total, trues and NECR peak rate of (1141.59 ± 1.51) kcps@147.34 kBq/μL, (1026.14 ± 0.43) kcps@147.34 kBq/μL, and (922.37 ± 1.35) kcps@147.34 kBq/μL, respectively, when the two half-cylinders are closed. In accordance with the sensitivity, the count rate decreases of approximately 30% when the half-cylinders are displaced (Figures 4D,E). More interestingly, the count rate increases of an approximate factor of 4 when the extended energy window Δ*E*(*ext*) is used (Figures 4F–H). For instance, we expect a total, trues and NECR peak rate of (3690.27 ± 2.81) kcps@157.19 kBq/μL, (3269.37 ± 2.56) kcps@147.34 kBq/μL, and (2705.69 ± 2.33) kcps@147.34 kBq/μL, respectively, when the two half-cylinders are closed. The count rate performance is summarized in the Supplementary Table 2.

The scatter fraction is approximately 4% when using the traditional energy window and never exceeds approximately 11% when enlarging the energy window, as reported in the Supplementary Table 3. This indicates that the probability of Compton scattering in plants is very small and confirms the feasibility of the extension of the energy selection window in plant PET applications, with a remarkable improvement of the sensitivity without negatively affecting the NECR of the system.

#### 3.1.3. Spatial Resolution

A summary of the spatial resolution of the system at the center of the FOV for different separations between the half-cylinders and different energy windows is reported in Figure 4I. When the half-cylinders are closed and the source is positioned in the FOV center, the axial, radial and tangential spatial resolutions are 1.90 ± 0.60 mm, 1.38 ± 0.19 mm, and 1.38 ± 0.19 mm, respectively. When increasing the energy window, these values increase up to 2.17 ± 0.91 mm, 1.75 ± 0.13 mm, and 1.75 ± 0.13 mm, respectively. The separation between the half-cylinders has a significant effect on the radial spatial resolution, which increases up to 3.43 ± 0.66 mm at the center of the FOV for a separation of 40 mm. As summarized in the Supplementary Tables 4–7, a slight degradation of the axial spatial resolution is also observed at 1/4 of the longitudinal FOV. As for the dependence of the spatial resolution on the position in the transverse plane, only the tangential spatial resolution has a pronounced dependence on the position on the X-axis, deteriorating up to 6.30 ± 2.15 mm at a distance of 35 mm from the center for the FOV. Similarly, only the radial spatial resolution has a pronounced dependence on the position of the Y-axis, deteriorating up to 6.85 ± 0.99 mm at a distance of 35 mm from the center for the FOV.

### 3.2. Miniaturized System

#### 3.2.1. Sensitivity

The estimated sensitivity of the miniaturized systems is shown in Figure 5A for the conventional energy window Δ*E*(*trad*) and in Figure 5B for the extended energy window Δ*E*(*ext*). Similarly to the **CONC** systems, its value reaches a maximum at the center of the FOV and decreases linearly following the typical expectations due to translational axial symmetry. When the half-cylinders are closed and the energy window Δ*E*(*trad*) is used, the peak sensitivity ranges between (1.057 ± 0.007)% and (0.763 ± 0.006)% for a crystal length of 20 and 13 mm, respectively. When the half-cylinders are displaced from each other, the reduced solid angle coverage causes up to 50% decrease of the sensitivity. The extension of the energy window is beneficial. We observe that when the half-cylinders are closed and the energy window Δ*E*(*ext*) is used, the peak sensitivity reaches a value of (12.861 ± 0.027)%, thus exhibiting an almost 10-fold gain with respect to Δ*E*(*trad*). The sensitivity increases of approximately 50% when the crystal length is expanded from 13 mm to 20 mm.

#### 3.2.2. Count Rate Performance

The count rate performance of the miniaturized systems in a conventional configuration, with closed half-cylinders and small energy window Δ*E*(*trad*) is shown in Figure 5C. The highest values are recorded for the **MINI-20** system, when the two half-cylinders are closed, with a total, trues and NECR peak rate of (301.55 ± 0.77) kcps@353.68 kBq/μL, (239.68 ± 0.69) kcps@353.68 kBq/μL, and (190.92 ± 0.69) kcps@353.68 kBq/μL, respectively. In accordance with the sensitivity, the count rate decreases of approximately 50% when the half-cylinders are displaced (Figures 5D,E). More interestingly, the count rate increases approximately 10 times when the extended energy window Δ*E*(*ext*) is used (Figures 5F–H). For instance, we expect a total, trues and NECR peak rate of (2944.85 ± 2.43) kcps@353.68 kBq/μL, (1789.68 ± 1.89) kcps@353.68 kBq/μL, and (1087.64 ± 1.70) kcps@353.68 kBq/μL, respectively, when the two half-cylinders are closed. A summary of the count rate characterization is reported in the Supplementary Table 8.

The scatter fraction is smaller than 10% when using the traditional energy window and never exceeds approximately 37% when enlarging the energy window, as reported in the Supplementary Table 9.

#### 3.2.3. Spatial Resolution

A summary of the spatial resolution of the system at the center of the FOV for different separations between the half-cylinders, different energy windows and crystal lengths is reported in Figure 5I. When the half-cylinders are closed and the source is positioned in the FOV center, the axial, radial and tangential spatial resolutions are 1.06 ± 0.05 mm, 1.00 ± 0.03 mm, and 1.00 ± 0.03 mm, respectively. When increasing the energy window, these values increase up to 1.14 ± 0.06 mm, 1.11 ± 0.04 mm, and 1.12 ± 0.04 mm, respectively. The separation between the half-cylinders has a significant effect on the radial spatial resolution, which increases up to 1.88 ± 0.41 mm at the center of the FOV for a separation of 40 mm. As summarized in the Supplementary Tables 10–13, a slight degradation of the axial spatial resolution is also observed at 1/4 of the longitudinal FOV. As for the dependence of the spatial resolution on the position in the transverse plane, only the tangential spatial resolution has a pronounced dependence on the position on the X-axis, deteriorating up to 4.43 ± 0.87 mm at a distance of 10 mm from the center of the FOV. Similarly, only the radial spatial resolution has a pronounced dependence on the position of the Y-axis, deteriorating up to 9.25 ± 0.51 mm at a distance of 10 mm from the center for the FOV. The crystal length does not seem to have a significant effect on the spatial resolution.

### 3.3. De Renzo Imaging

We can appreciate in Figure 6 the results of the De Renzo phantom reconstruction in three different radial positions. They evidence the a good resolution of the holes with a diameter of 2.2 mm and 2.0 mm for all the displacements of the phantom, even when their relative distance of the holes from the center of the FOV is >4.5 mm. The resolution of the holes with 1.6 mm and 1.8mm diameters is poor as expected but it increases when their relative distance from the center of the FOV is reduced. It is clear that, around the center of the **CONC** system in a 20mm opening configuration, source distributions within those small volumes are difficult to distinguish outside a range of few millimeters.

### 3.4. Plant Phantom Imaging

Figures 7A,B report the values in percentage over 1 million simulated events for each evaluated parameter for ^18^F and ^11^C radionuclides. We estimated that 2.61% and 2.35% of the interactions in the plant-like phantom are primary Compton processes, respectively.

Such percentages correspond to the the number of events generating γ-rays that interact with the vegetal tissue and releasing part of their original energy and momentum via Compton scattering. The total number of events with an interaction in the plant phantom, including photoelectric processes, rises up to 4.91% for ^18^F and 4.83% for ^11^C. Instead, the number of escaped positrons from the original volume is 0.05% for ^18^F and 0.34% for ^11^C. Among the simulated plastic phantoms, PLA performances are close to the simulated plant phantom made of water and cellulose. A typical simulation framework and reconstructed image of the simulated phantom is shown in Figures 7C,D. The section of the phantom reproducing the stem and the vascular system are well visible. A 3D movie of the reconstructed image from many different angles can be found in the Supplementary Material.

## 4. Discussion

The findings presented here can be benchmarked with respect to existing plant PET systems. The **CONC** design addresses the needs of an extended longitudinal and transverse FOV for dedicated plant imaging, without overloading sensors technology. The FOV adjustability is the most striking feature of the novel design. The axial FOV, oriented vertically, is 100 mm and the transverse FOV is oval-shaped, with a minimal diameter of 83.4 mm, extendible up to 123.4 mm. These values are in good agreement with the Open PET system, which has an axial and transverse FOV of 110 mm and up to 126 mm, respectively (Yamaya et al., 2011). The PETIS system has a larger transverse and axial FOV, but the planar geometry allows only 2D imaging (Kawachi et al., 2006).

The **CONC** design is not particularly demanding from a technological point of view. The cross section of the crystals is in fact 3.95 mm and is larger than in existing plant PET systems, where it ranges between 1.5 mm (Wang et al., 2014) and 2.9 mm (Yamaya et al., 2011). It determines the minimal reachable spatial resolution of 1.38 mm at the center of the FOV. This value is still competitive with respect to the spatial resolution obtained in other plant PET systems, which is ranging between 1.25 mm (Wang et al., 2014) and 2.3 mm (Kawachi et al., 2006). We observe a series of characteristic features when the transverse FOV is extended by displacing the two half-cylinders and acquires an oval shape. As visible in Figure 4I, in fact, a degradation of the radial spatial resolution is expected as a consequence of the displacement of the half-cylinders. More interestingly, the depth of interaction error affects the transverse components of the spatial resolution differently, when the transverse FOV is extended. In particular, the radial and the tangential spatial resolution are adversely worsened along the minor and major transverse axis, respectively.

A comparison between the NEMA characterization of the spatial resolution and the De Renzo imaging provides also a better view of the achievable image quality. We considered the CONC prototype with a 20 mm opening. As shown in Figure 6, De Renzo imaging reveals a good discrimination power of the holes down to 1.8 mm, which is even better than the single point spatial resolution. In fact, from the NEMA evaluation on the **CONC** system in a 20 mm opened configuration in the Δ*E*(*ext*) window regime, we can appreciate a FWHM from 2.04 mm at the center of the FOV to 2.56 and 2.77 mm when the source is radially displaced 5 and 10mm, respectively (Figure 4I). It is evident that it is possible to provide a good spatial resolution also for distributed sources in an oval configuration, encouraging the pioneering idea of shape-adaptability for plant PET cameras.

The miniaturization of the system by using smaller section crystals allows to obtain a potential competitive spatial resolution of approximately 1 mm. We report similar effects to the axial, tangential and radial spatial resolutions in the **CONC** and **MINI** designs, due to the oval-shaped FOV. Figure 5I, in addition, reveals that the contribution of the crystal length to the spatial resolution is negligible with respect to other systematic sources. Such a compact design is in fact affected by cracks between PET heads and a significant DOI error, which need to be modeled with a proper image reconstruction. It may be mandatory therefore to include the Point Spread Function calculation in the OSEM algorithm in order to have a more precise estimation of the spatial resolution, which is expected to reach a sub-millimetric level (D'Ascenzo et al., 2018). The **MINI** design is conceived for the imaging of small sprouts, with fast portability to the greenhouse, and has a FOV significantly smaller than other PET systems. This is not a limitation of the system. The modular structure allows in fact to extend the FOV as desired.

It is evident that the smaller crystal pitch is preferable, in order to achieve a competitive spatial resolution. However, the number of readout channels of the system increases together with the readout complexity and the related dead time. This is particularly important when considering the sensitivity and the NECR of the systems. As shown in Figures 4A,B, 5A,B, the peak value of the **CONC** and **MINI** system sensitivities is 8% and 1%, respectively. As we verified in the phantom study (Figures 7A,B), the probability of Compton scattering and attenuation in the plant tissue is approximately 4%. In D'Ascenzo et al. (2020), using the sensor technology we based our **CONC** design, the probability of Compton scatter in a mouse phantom made of PMMA and with a 25 mm diameter and 70 mm length is approximately 8%. It is therefore reasonable, even considering larger or denser plant samples, to extend the energy window of PET imaging to a larger range Δ*E*(*ext*) = (50, 750) keV. When using Δ*E*(*ext*), the sensitivity of the system has a sizable increase up to a peak value of 41% and 13% in the *CONC* and *MINI* systems, respectively. These values are competitive with respect to other plant PET systems, which report a sensitivity between 1.3% (Wang et al., 2014) and 8.7% (Yamaya et al., 2011). The estimated SF confirms our expectations. It increases from 4% when a conservative energy window is used up to 11% and 30% in the **CONC** and **MINI** systems, respectively, when Δ*E*(*ext*) is used. This shows that a certain level of inter-crystal scattering occurs in the systems even at low activities and generates a large number of random coincidences at high activities (Figures 4C–H, 5C–H), which can be easily suppressed with dedicated coincidence schemes. However, we may note that the spatial resolution of both systems is not affected significantly by the expansion of the energy window.

Even if the sensitivity decreases when the half-cylinders get displaced from each other, its value is never below 10% when Δ*E*(*ext*) is used. This implies an expected count rate of up to 40 Mcps. Such high sensitivity plant PET system will therefore require a large bandwidth readout system, which is possible thanks to novel digital technologies (D'Ascenzo et al., 2018, 2020).

Positron escape plays an important role in plant PET imaging, due to the thin and soft plant structures. According to Alexoff et al. (2011), the expected amount of escaped positrons for a simulated plant leaf with a 2 mm thickness is around 13%, while in our simulation we record that only the 0.2% of positrons do not annihilate in the phantom volume. We can address such discrepancy to the fact that we involved in the analysis the stem of our designed scatter phantom, that is 50% thicker than the leaf, consists in the 83% of the entire volume and the source distribution is confined in a 0.5 mm hole along the longitudinal axis for radiotracer injection reasons. It may be therefore reasonable to provide a proper “leaf phantom” in order to quantify the effect of positron escape in plant PET imaging. In literature, Partelová et al. (2016) described how to cast discs imitating plant tissues 0.8 mm thick by using ^18^F-enriched agar solution. Although that solution returns very thin phantoms already loaded with radiotracer, it is likely to have a not negligible portion of radiotracer directly on the phantom surface, making the phantom not suitable as such for positron escape analysis. However, providing a thin shell to Partelová et al. (2016) leaf phantom may return more reliable results and that can be matter for further works.

Finally, a key result of this study is the definition of a set of new standards for the evaluation of plant PET systems, which can be used in the future in order to compare plant PET scanners in a way, which is more suited to the agronomic perspective. The results in Figures 7A,B suggest that in a plant phantom composed of PLA is to be preferred, in order to carry the characterization procedures proposed in the paper.

## 5. Conclusions

As recently noticed, Plant Positron Emission Tomography is an emerging field of research, which requires a strong cross-disciplinary interplay between physics, engineering, mathematics, biology, and agronomy (Mincke et al., 2021). A first result of this study is to determine the method of communication between these disciplines during the design and optimization of a plant PET system, on the basis of a standard assessment strategy. We therefore extended and re-adapted to agronomy the typical procedures of clinical PET design. In particular, the design proposed in our paper is at the basis of the currently ongoing realization of a portable plant PET system.

Beyond these novel methodological aspects, the design study proposed in this paper highlighted the theoretical basis of the technological challenges posed by plant PET, which include high sensitivity and count rate performance, in order to detect the weak signals from the soft and thin plant tissues. In addition, we demonstrated that the compact and shape-adaptable geometry of a plant PET system introduce the problems of DOI error and limited angle availability, which reflects to the uniformity of the spatial resolution in the extendible FOV. These challenges are recently driving new CMOS-based sensor technologies (D'Ascenzo et al., 2017), signal processing methods and dedicated image reconstruction algorithms, which will be instrumental to achieve the required precision for quantitative *in-vivo* functional measurements in digital agriculture.

## Data Availability Statement

The raw data supporting the conclusions of this article will be made available by the authors, without undue reservation.

## Author Contributions

EA, ND'A, and MP analyzed the data and wrote the manuscript. MB performed the MINI system simulation. EA, DC, and ND'A designed the plant PET systems. CK performed the CONC system simulation. MC, GP, and MP designed the possible applications to agronomy. JW, SW, and FZ developed the image reconstruction algorithms. ND'A, MP, and QX supervised the research focus and designed the scientific background. GR provided inputs on portable systems. LG was responsible of the computing platform. All authors contributed to the article and approved the submitted version.

## Funding

We acknowledge the support of the Horizon 2020 Research and Innovation Staff Exchange (RISE) Call: H2020-MSCA-RISE-2020 (101008114); Sino-German science center NSFC/DFG (M-0387); the National Key Research and Development Program of the People's Republic of China Key Program for Intergovernmental Cooperation in International Science and Technology Innovation (2018YFE0118900); National R&D Program for Major Research Instruments of Natural Science Foundation of China (61927801); National R&D Program for Major Research Instruments of Natural Science Foundation of China (6027808); National Key Research and Development of China (2019YFC0118900); the original exploration project recommended by experts of the special project of National Natural Science Foundation of China (62050288); the International Graduate School MEMoRIAL funded by the European Social Fund Profram (Sachsen-Anhalt Wissenschaft Internationalisierung).

## Conflict of Interest

The authors declare that the research was conducted in the absence of any commercial or financial relationships that could be construed as a potential conflict of interest.

## Publisher's Note

All claims expressed in this article are solely those of the authors and do not necessarily represent those of their affiliated organizations, or those of the publisher, the editors and the reviewers. Any product that may be evaluated in this article, or claim that may be made by its manufacturer, is not guaranteed or endorsed by the publisher.
