# Supplementary material for: Design Study of a Novel Positron Emission Tomography System for Plant Imaging
Source: Front Plant Sci. 2022 Jan 18;12:736221. doi: 10.3389/fpls.2021.736221 (PMC8805640; doi:10.3389/fpls.2021.736221)
Supplement: Supplementary file 1 [file Data_Sheet_1.PDF]

Supplementary tables

Conceptual System

Table 1: Count Rate performance simulation setup.

Frame	^{18}F activity (MBq)	^{18}F simulated events
0	150.0	75,000,000
1	141.7	70,800,000
2	133.3	66,700,000
3	125.0	62,500,000
4	116.7	58,300,000
5	108.3	54,200,000
6	100.0	50,000,000
7	91.7	45,800,000
8	83.3	41,700,000
9	75.0	37,500,000
10	68.3	34,100,000
11	61.6	30,800,000
12	54.9	27,400,000
13	48.2	24,100,000
14	41.4	20,700,000
15	34.7	17,400,000
16	28.0	14,000,000
17	21.3	10,700,000
18	14.6	7,300,000
19	7.9	3,900,000
20	1.2	600,000

Table 2: Conceptual system: summary of the count rate performance

opening	total [kcps]	trues [kcps]	NEC [kcps]
Energy window $\Delta E\,(trad) = (350, 650)$ keV			
0 mm	1141.59 ±1.51	1026.14 ±.43	922.37 ±1.35
20 mm	1048.55 ±1.45	933.2 ±1.36	830.54 ±1.28
40 mm	880.5 ±1.32	775.76 ±1.24	683.47 ±1.16
Energy window $\Delta E\,(ext) = (50, 750)$ keV			
0 mm	3960.27 ±2.81	3269.37 ±2.56	2705.69 ±2.33
20 mm	3611.71 ±2.69	2971.07 ±2.43	2444.06 ±2.21
40 mm	3051.48 ±2.47	2504.08 ±2.23	2054.87 ±2.02

Table 3: Conceptual system: scatter fraction.

opening	SF ($\Delta E\,(trad)$) [%]	SF ($\Delta E\,(ext)$) [%]
0 mm	4.1 ±0.18	10.9 ±0.05
20 mm	4.0 ±0.24	10.5 ±0.06
40 mm	5.7 ±0.32	11.3 ±0.08

Table 4: Conceptual system: spatial resolution sampled on the X axis in the transverse plane. The traditional energy range $\Delta E\,(trad)$ is used.

opening	FOV	position [mm]	axial [mm]	radial [mm]	tangential [mm]
0 mm	centre	0	1.90 ±0.60	1.38 ±0.19	1.38 ±0.19
		5	1.87 ±0.70	1.97 ±0.30	2.35 ±0.32
		10	1.99 ±0.67	2.11 ±0.39	3.64 ±0.47
		15	2.00 ±0.83	2.35 ±0.62	2.59 ±0.64
		25	1.82 ±0.74	2.93 ±0.53	4.24 ±0.97
		35	1.66 ±0.64	3.04 ±0.52	6.43 ±1.20
	one-fourth	0	2.42 ± 1.6	1.37 ± 0.18	1.37 ± 0.18
		5	2.44 ± 1.3	1.93 ± 0.27	2.37 ± 0.31
		10	2.65 ± 2.1	2.14 ± 0.37	3.68 ± 0.57
		15	3.11 ± 1.97	2.36 ± 0.60	2.57 ± 0.63
		25	2.59 ± 1.52	2.85 ± 0.51	4.28 ± 0.83
		35	2.46 ± 1.04	2.99 ± 0.50	6.10 ± 1.25
20 mm	centre	0	1.83 ± 0.51	1.62 ±0.25	1.38±0.22
		5	1.82 ±0.60	2.06 ± 0.55	2.26±0.38
		10	1.90 ± 0.56	2.30 ± 0.57	2.83 ±0.65
		15	1.95 ± 0.70	2.56 ±0.78	2.87 ±0.81
		25	1.72 ± 0.60	3.05 ±0.93	4.09 ±1.16
		35	1.50 ±0.48	3.88 ±1.49	6.37 ±2.49
	one-fourth	0	2.25 ±1.28	1.61 ±0.25	1.38±0.22
		5	2.28 ±1.06	2.03 ±0.47	2.23±0.36
		10	2.55 ±1.66	2.26 ±0.56	2.86 ±0.65
		15	2.68 ±1.70	2.54 ±0.75	2.87 ±0.79
		25	2.41 ±1.54	2.98 ±0.79	3.98 ±1.16
		35	2.17 ±0.63	3.82 ±1.40	6.64 ±2.61
40 mm	centre	0	1.70 ±0.44	2.21 ±0.51	1.41 ±0.21
		5	1.68 ±0.51	2.57 ±0.61	2.29 ±0.45
		10	1.83 ±0.49	3.02 ±0.78	2.88 ±0.71
		15	1.83 ±0.56	3.29 ±0.94	2.62 ±0.77
		25	1.63 ±0.49	4.40 ±1.57	4.24 ±1.23
		35	1.47 ±0.38	5.10 ±2.72	6.72 ±1.66
	one-fourth	0	2.18 ±0.93	2.19 ±0.51	1.41 ±0.21
		5	2.08 ±1.31	2.49 ±0.61	2.34 ±0.43
		10	2.43 ±1.46	2.97 ±0.68	2.84 ±0.69
		15	2.53 ±1.55	3.20 ±0.91	2.63 ±0.77
		25	2.31 ±1.19	4.20 ±1.49	4.11 ±1.24
		35	1.98 ±0.44	4.92 ±1.98	6.77 ±1.65

Table 5: Conceptual system: spatial resolution sampled on the Y axis in the transverse plane. The traditional energy range $\Delta E\,(trad)$ is used.

opening	FOV	position [mm]	axial [mm]	radial [mm]	tangential [mm]
0 mm	centre	0	1.88 ±0.90	1.39 ±0.13	1.38 ±0.13
		5	1.83 ±1.10	2.08 ±0.36	1.78 ±0.33
		10	1.97 ±1.02	2.89 ±0.88	2.01 ±0.40
		15	1.92 ±1.11	2.56 ±0.70	2.10 ±0.48
		25	1.83 ±1.12	3.55 ±1.19	2.25 ±0.54
		35	1.76 ±1.00	6.67 ±2.58	2.77 ±0.89
	one-fourth	0	2.42 ±1.58	1.37 ±0.12	1.37 ±0.12
		5	2.55 ±3.58	2.04 ±0.34	1.78 ±0.31
		10	2.64 ±2.10	3.02 ±0.89	2.01 ±0.38
		15	2.76 ±2.30	2.52 ±0.67	2.10 ±0.47
		25	2.67 ±1.64	3.56 ±1.16	2.23 ±0.52
		35	2.62 ±1.40	6.76 ±2.81	2.70 ±0.88
20 mm	centre	0	1.83 ±0.75	1.63 ±0.22	1.38 ±0.14
		5	1.77 ±0.95	1.87 ±0.36	1.61 ±0.32
		10	2.02 ±0.94	2.58 ±0.61	2.12 ±0.41
		15	2.02 ±1.04	3.30 ±1.02	1.91 ±0.48
		25	1.78 ±0.94	3.25 ±1.86	2.55 ±0.77
		35	1.83 ±0.94	4.29 ±2.61	2.44 ±0.54
	one-fourth	0	2.34 ±1.31	1.60 ±0.22	1.38 ±0.14
		5	2.38 ±3.02	1.86 ±0.35	1.59 ±0.32
		10	2.74 ±1.79	2.61 ±0.63	2.09 ±0.40
		15	2.99 ±3.13	3.36 ±1.05	1.89 ±0.47
		25	2.55 ±1.56	3.17 ±1.36	2.55 ±0.76
		35	2.59 ±1.29	4.46 ±2.63	2.44 ±0.54
40 mm	centre	0	1.70 ±0.65	2.19 ±0.28	1.40 ±0.14
		5	1.71 ±0.83	2.44 ±0.46	1.53 ±0.30
		10	1.89 ±0.78	3.57 ±0.95	2.17 ±0.40
		15	2.04 ±0.97	3.09 ±0.92	1.81 ±0.42
		25	1.75 ±0.74	3.98 ±1.75	2.17 ±0.54
		35	1.66 ±0.84	3.59 ±2.42	2.00 ±0.58
	one-fourth	0	2.55 ±0.90	4.18 ±0.29	2.16 ±0.13
		5	2.07 ±1.03	2.54 ±0.71	1.55 ±0.30
		10	2.41 ±1.37	3.79 ±0.96	2.16 ±0.39
		15	2.68 ±1.52	3.09 ±0.93	1.79 ±0.41
		25	2.55 ±1.26	4.18 ±1.82	2.17 ±0.55
		35	2.35 ±1.11	3.66 ±2.48	1.95 ±0.56

Table 6: Conceptual system: spatial resolution sampled on the X axis in the transverse plane. The extended energy range $\Delta E\,(ext)$ is used.

opening	FOV	position [mm]	axial [mm]	radial [mm]	tangential [mm]
0 mm	centre	0	2.17 ±0.91	1.75 ±0.13	1.75 ±0.13
		5	2.15 ±1.05	2.37 ±0.33	2.79 ±0.37
		10	2.26 ±0.97	2.52 ±0.39	4.16 ±0.60
		15	2.27 ±1.24	2.89 ±0.59	3.07 ±0.83
		25	2.15 ±1.09	3.36 ±0.58	4.79 ±1.41
		35	2.07 ±0.97	3.43 ±0.66	6.30 ±2.15
	one-fourth	0	3.01 ±1.58	1.74 ±0.12	1.73 ±0.12
		5	3.41 ±3.07	2.33 ±0.30	2.78 ±0.36
		10	3.32 ±1.95	2.53 ±0.37	4.19 ±0.58
		15	3.39 ±3.76	2.86 ±0.58	3.08 ±0.82
		25	2.95 ±1.56	3.26 ±0.65	4.81 ±1.41
		35	2.83 ±1.27	3.38 ±0.63	6.30 ±5.69
20 mm	centre	0	2.13 ±0.76	2.04 ±0.22	1.74 ±0.14
		5	2.09 ±0.89	2.56 ±0.34	2.71 ±0.43
		10	2.20 ±0.79	2.77 ±0.41	3.66 ±1.14
		15	2.26 ±1.01	3.17 ±0.84	3.37 ±1.02
		25	2.09 ±0.85	3.65 ±0.81	4.69 ±2.12
		35	1.90 ±0.53	4.85 ±1.31	5.95 ±4.23
	one-fourth	0	2.85 ±1.24	2.02 ±0.22	1.73 ±0.14
		5	3.29 ±2.61	2.50 ±0.34	2.67 ±0.42
		10	3.11 ±1.47	2.74 ±0.40	3.66 ±1.14
		15	3.46 ±2.14	3.10 ±0.73	3.39 ±0.98
		25	2.93 ±1.40	3.57 ±0.79	4.65 ±1.89
		35	2.57 ±0.74	4.66 ±1.22	6.13 ±4.23
40 mm	centre	0	2.03 ±0.65	2.70 ±0.28	1.77 ±0.14
		5	1.99 ±0.75	3.20 ±0.55	2.77 ±0.48
		10	2.12 ±0.71	3.59 ±0.59	3.66 ±0.79
		15	2.15 ±0.80	4.00 ±0.75	3.15 ±0.71
		25	2.00 ±0.72	5.54 ±1.43	4.70 ±1.58
		35	1.81 ±0.32	6.75 ±1.87	6.70 ±2.65
	one-fourth	0	2.70 ±0.94	2.70 ±0.27	1.77 ±0.14
		5	2.69 ±0.95	3.17 ±0.41	2.79 ±0.47
		10	2.99 ±1.27	3.51 ±0.57	3.65 ±0.83
		15	3.21 ±1.58	3.88 ±0.72	3.21 ±0.70
		25	2.86 ±1.27	5.30 ±1.23	4.60 ±1.64
		35	2.36 ±0.66	6.38 ±1.73	6.81 ±2.62

Table 7: Conceptual system: spatial resolution sampled on the Y axis in the transverse plane. The extended energy range $\Delta E\,(ext)$ is used.

opening	FOV	position [mm]	axial [mm]	radial [mm]	tangential [mm]
0 mm	centre	0	2.16 ±0.59	1.76 ±0.19	1.75 ±0.19
		5	2.13 ±0.71	2.59 ±0.33	2.16 ±0.29
		10	2.23 ±0.69	3.44 ±0.76	2.37 ±0.34
		15	2.21 ±0.75	3.07 ±0.77	2.53 ±0.55
		25	2.17 ±0.77	3.93 ±0.66	2.71 ±0.55
		35	2.14 ±0.66	6.85 ±0.99	3.29 ±0.72
	one-fourth	0	3.00 ±1.60	1.74 ±0.18	1.73 ±0.18
		5	3.46 ±1.27	2.56 ±0.32	2.15 ±0.27
		10	3.26 ±2.32	3.56 ±0.73	2.38 ±0.33
		15	3.26 ±1.80	3.08 ±0.75	2.52 ±0.47
		25	3.11 ±1.54	3.92 ±0.64	2.65 ±0.54
		35	2.91 ±1.11	6.87 ±0.98	3.21 ±0.69
20 mm	centre	0	2.14 ±0.51	2.04 ±0.26	1.73 ±0.22
		5	2.08 ±0.62	2.28 ±0.38	1.98 ±0.30
		10	2.30 ±0.65	3.04 ±0.78	2.50 ±0.46
		15	2.29 ±0.72	3.65 ±0.85	2.28 ±0.43
		25	2.11 ±0.65	3.59 ±1.13	3.07 ±0.71
		35	2.15 ±0.64	4.37 ±1.32	2.84 ±0.54
	one-fourth	0	2.85 ±1.30	2.02 ±0.25	1.74 ±0.22
		5	3.40 ±1.13	2.27 ±0.38	1.97 ±0.29
		10	3.42 ±1.98	3.09 ±0.77	2.46 ±0.38
		15	3.37 ±1.70	3.66 ±0.87	2.25 ±0.42
		25	3.04 ±1.51	3.54 ±1.14	3.04 ±0.70
		35	2.96 ±1.06	4.38 ±1.30	2.80 ±0.54
40 mm	centre	0	2.03 ±0.44	2.68 ±0.51	1.76 ±0.21
		5	2.02 ±0.56	3.15 ±0.79	1.92 ±0.28
		10	2.02 ±0.56	3.15 ±0.95	1.92 ±0.46
		15	2.31 ±0.69	3.65 ±1.07	2.19 ±0.39
		25	2.09 ±0.52	4.20 ±1.33	2.65 ±0.53
		35	2.01 ±0.56	3.61 ±1.52	2.41 ±0.48
	one-fourth	0	2.66 ±0.92	2.70 ±0.51	1.78 ±0.21
		5	3.27 ±0.95	3.28 ±0.80	1.93 ±0.28
		10	2.91 ±2.49	4.36 ±0.99	2.60 ±0.46
		15	3.28 ±1.75	3.68 ±1.07	2.17 ±0.38
		25	2.91 ±0.97	4.22 ±1.28	2.62 ±0.53
		35	2.68 ±0.73	3.63 ±1.56	2.36 ±0.47

Miniaturized System

Table 8: Miniaturized system: summary of the count rate performance

crystal size	opening	total [kcps]	trues [kcps]	NEC [kcps]
Energy window $\Delta E\,(trad) = (350, 650)$ keV				
13 mm	0 mm	223.84 ±0.67	179.48 ±0.60	143.92 ±0.61
	20 mm	110.79 ±0.47	81.35 ±0.40	51.74 ±0.34
	40 mm	73.28 ±0.38	49.78 ±0.31	33.82 ±0.26
16 mm	0 mm	261.01 ±0.72	207.66 ±0.64	165.21 ±0.65
	20 mm	177.74 ±0.60	129.63 ±0.51	94.54 ±0.50
	40 mm	116.59 ±0.48	78.11 ±0.39	52.33 ±0.37
20 mm	0 mm	301.55 ±0.77	239.68 ±0.69	190.50 ±0.69
	20 mm	210.66 ±0.65	146.49 ±0.54	101.87 ±0.52
	40 mm	139.93 ±0.53	89.55 ±0.42	57.30 ±0.40
Energy window $\Delta E\,(ext) = (50, 750)$ keV				
13 mm	0 mm	2220.21 ±2.10	1362.32 ±1.65	835.914 ±1.49
	20 mm	1051.73 ±1.45	628.95 ±1.12	376.12 ±0.86
	40 mm	675.64 ±1.16	359.06 ±0.84	190.82 ±0.61
16 mm	0 mm	2580.65 ±2.27	1579.96 ±1.77	967.30 ±1.60
	20 mm	1697.75 ±1.84	981.83 ±1.40	567.81 ±1.22
	40 mm	1099.26 ±1.48	559.23 ±1.06	284.50 ±0.85
20 mm	0 mm	2944.85 ±2.43	1789.68 ±1.89	1087.64 ±1.70
	20 mm	1999.19 ±2.00	1101.65 ±1.48	607.06 ±1.25
	40 mm	1326.41 ±1.62	637.01 ±1.12	305.92 ±0.87

Table 9: Miniaturized systems: scatter fraction.

opening	SF ($\Delta E\,(trad)$) [%]	SF ($\Delta E\,(ext)$) [%]	
13 mm	0 mm	3.87 ±0.12	27.4 ±0.09
	20 mm	2.6 ±4.63	29.4 ±0.27
	40 mm	4.3 ±7.10	31.3 ±0.42
16 mm	0 mm	4.72 ±0.12	28.79 ±0.08
	20 mm	7.20 ±0.19	32.25 ±0.12
	40 mm	7.50 ±0.25	33.09 ±0.15
20 mm	0 mm	6.36 ±0.13	29.96 ±0.08
	20 mm	10.16 ±0.21	35.75 ±0.11
	40 mm	9.56 ±0.25	36.80 ±0.14

Table 10: Miniaturized system: spatial resolution sampled on the X axis in the transverse plane. The traditional energy range $\Delta E\,(trad)$ is used.

opening	FOV	position [mm]	axial [mm]	radial [mm]	tangential [mm]
0 mm	centre	0	1.06 ±0.05	1.00 ±0.03	1.00 ±0.03
		1	1.02 ±0.07	1.06 ±0.06	0.83 ±0.17
		2	1.07 ±0.08	1.12 ±0.08	1.35 ±0.09
		3	1.10 ±0.08	1.10 ±0.07	1.02 ±0.32
		5	1.09 ±0.09	1.28 ±0.12	2.45 ±0.32
		10	1.12 ±0.09	1.77 ±0.18	3.06 ±0.33
	one-fourth	0	1.94 ±0.11	1.01 ±0.04	1.01 ±0.04
		1	1.25 ±0.12	1.05 ±0.06	0.80 ±0.07
		2	2.03 ±0.12	1.10 ±0.07	1.36 ±0.08
		3	2.03 ±0.11	1.06 ±0.06	1.25 ±0.36
		5	2.15 ±0.10	1.29 ±0.10	2.49 ±0.40
		10	2.30 ±0.08	1.73 ±0.17	3.31 ±0.34
20 mm	centre	0	1.05 ±0.03	1.38 ±0.34	1.32 ±0.06
		1	1.04 ±0.04	1.58 ±0.14	0.63 ±0.02
		2	1.04 ±0.04	2.01 ±0.10	1.63 ±0.07
		3	1.04 ±0.04	1.46 ±0.10	1.72 ±0.11
		5	1.07 ±0.05	1.77 ±0.08	2.55 ±0.15
		10	1.07 ±0.05	2.87 ±0.34	3.26 ±0.15
	one-fourth	0	1.52 ±0.07	3.09 ±0.06	1.34 ±0.06
		1	1.47 ±0.07	1.73 ±0.12	0.65 ±0.02
		2	0.98 ±0.06	2.15 ±0.13	1.43 ±0.06
		3	1.42 ±0.07	1.44 ±0.09	1.69 ±0.11
		5	1.71 ±0.09	1.81 ±0.07	2.63 ±0.11
		10	1.24 ±0.16	2.27 ±0.31	3.49 ±0.13
40 mm	centre	0	1.05 ±0.03	1.66 ±0.39	1.06 ±0.03
		1	1.02 ±0.03	2.10 ±0.20	0.69 ±0.03
		2	1.03 ±0.03	2.30 ±0.13	1.58 ±0.05
		3	1.00 ±0.03	2.26 ±0.13	2.16 ±0.07
		5	1.06 ±0.03	2.89 ±0.15	3.32 ±0.20
		10	1.01 ±0.04	3.34 ±0.28	2.89 ±0.54
	one-fourth	0	1.02 ±0.05	6.12 ±0.26	1.07 ±0.03
		1	1.19 ±0.07	5.05 ±0.44	0.72 ±0.03
		2	0.89 ±0.09	2.40 ±0.13	1.66 ±0.05
		3	0.92 ±0.05	2.62 ±0.12	2.39 ±0.05
		5	1.52 ±0.07	3.21 ±0.15	3.13 ±0.11
		10	1.05 ±0.13	2.97 ±0.20	3.88 ±0.22

Table 11: Miniaturized system: spatial resolution sampled on the Y axis in the transverse plane. The traditional energy range $\Delta E\,(trad)$ is used.

opening	FOV	position [mm]	axial [mm]	radial [mm]	tangential [mm]
0 mm	centre	0	1.07 ±0.07	1.02 ±0.05	1.01 ±0.05
		1	1.09 ±0.09	0.86 ±0.10	1.04 ±0.07
		2	1.09 ±0.09	1.21 ±0.18	1.07 ±0.08
		3	1.08 ±0.09	1.81 ±0.22	1.12 ±0.09
		5	1.12 ±0.11	2.37 ±0.69	1.17 ±0.12
		10	1.10 ±0.10	2.97 ±0.13	1.59 ±0.35
	one-fourth	0	1.94 ±0.11	1.01 ±0.05	1.02 ±0.05
		1	1.78 ±0.11	0.82 ±0.08	1.06 ±0.07
		2	1.92 ±0.12	1.21 ±0.16	1.11 ±0.08
		3	2.03 ±0.11	1.71 ±0.22	1.11 ±0.08
		5	2.31 ±0.11	2.60 ±0.55	1.13 ±0.11
		10	2.00 ±0.11	3.00 ±0.28	1.49 ±0.29
20 mm	centre	0	1.03 ±0.04	1.36 ±0.41	1.23 ±0.08
		1	1.04 ±0.04	1.69 ±0.11	1.23 ±0.07
		2	1.04 ±0.04	1.91 ±0.06	1.17 ±0.08
		2	1.03 ±0.05	1.50 ±0.10	1.26 ±0.09
		5	1.10 ±0.07	2.53 ±0.10	1.18 ±0.07
		10	1.11 ±0.07	1.84 ±0.10	1.15 ±0.05
	one-fourth	0	1.40 ±0.07	2.22 ±0.07	1.34 ±0.07
		1	1.46 ±0.07	1.92 ±0.15	1.24 ±0.07
		2	1.00 ±0.07	2.01 ±0.05	1.18 ±0.06
		3	1.77 ±0.07	1.67 ±0.09	1.26 ±0.06
		5	1.47 ±0.10	2.71 ±0.23	1.24 ±0.06
		10	1.39 ±0.18	1.79 ±0.09	1.13 ±0.05
40 mm	centre	0	1.03 ±0.03	1.88 ±0.41	1.10 ±0.03
		1	1.07 ±0.03	1.60 ±0.25	1.08 ±0.04
		2	1.04 ±0.03	2.32 ±0.15	1.05 ±0.04
		3	1.02 ±0.03	2.43 ±0.27	1.09 ±0.04
		5	1.05 ±0.04	2.93 ±0.30	1.20 ±0.05
		10	1.08 ±0.05	1.51 ±0.14	1.07 ±0.04
	one-fourth	0	1.14 ±0.07	5.89 ±0.29	1.07 ±0.04
		1	1.11 ±.076	4.81 ±0.43	1.09 ±0.04
		2	0.99 ±0.10	3.13 ±0.26	1.13 ±0.04
		3	0.84 ±0.06	2.63 ±0.19	1.12 ±0.04
		5	1.64 ±0.07	2.99 ±0.20	1.18 ±0.04
		10	1.33 ±0.09	1.94 ±0.13	0.99 ±0.04

Table 12: Miniaturized system: spatial resolution sampled on the X axis in the transverse plane. The extended energy range $\Delta E\,(ext)$ is used.

opening	FOV	position [mm]	axial [mm]	radial [mm]	tangential [mm]
0 mm	centre	0	1.14 ±0.06	1.11 ±0.04	1.12 ±0.04
		1	1.12 ±0.08	1.20 ±0.07	1.09 ±0.07
		2	1.13 ±0.10	1.28 ±0.08	1.60 ±0.11
		3	1.16 ±0.10	1.26 ±0.09	1.87 ±0.13
		5	1.14 ±0.11	1.46 ±0.16	4.26 ±0.27
		10	1.17 ±0.14	2.09 ±0.29	4.43 ±0.87
	one-fourth	0	2.68 ±0.13	1.13 ±0.04	1.12 ±0.05
		1	2.75 ±0.19	1.20 ±0.06	1.03 ±0.06
		2	2.78 ±0.18	1.25 ±0.09	1.59 ±0.09
		3	2.82 ±0.18	1.22 ±0.07	1.79 ±0.17
		5	2.81 ±0.23	1.43 ±0.14	3.88 ±0.23
		10	2.76 ±0.34	2.05 ±0.24	4.55 ±0.52
20 mm	centre	0	1.16 ±0.03	2.13 ±0.05	1.61 ±0.05
		1	1.18 ±0.04	1.89 ±0.10	0.70 ±0.03
		2	1.15 ±0.04	2.62 ±0.25	1.75 ±0.06
		3	1.20 ±0.04	1.86 ±0.06	1.94 ±0.13
		5	1.20 ±0.05	1.96 ±0.11	2.92 ±0.13
		10	1.22 ±0.06	3.35 ±0.36	3.65 ±0.19
	one-fourth	0	1.71 ±0.08	3.98 ±0.09	1.66 ±0.05
		1	1.84 ±0.06	2.97 ±0.11	0.72 ±0.02
		2	1.77 ±0.06	2.56 ±0.28	1.62 ±0.05
		3	1.81 ±0.11	1.74 ±0.05	2.03 ±0.09
		5	1.85 ±0.10	1.99 ±0.09	2.92 ±0.10
		10	1.54 ±0.14	3.10 ±0.18	3.62 ±0.18
40 mm	centre	0	1.14 ±0.03	3.89 ±0.13	1.20 ±0.03
		1	1.17 ±0.03	2.66 ±0.09	0.80 ±0.03
		2	1.12 ±0.03	2.73 ±0.09	1.67 ±0.06
		3	1.11 ±0.03	2.54 ±0.07	2.46 ±0.09
		5	1.16 ±0.05	3.21 ±0.15	3.88 ±0.18
		10	1.15 ±0.05	3.88 ±0.59	3.94 ±0.50
	one-fourth	0	1.48 ±0.06	6.86 ±0.27	1.23 ±0.03
		1	1.61 ±0.05	5.02 ±0.31	0.82 ±0.03
		2	1.58 ±0.05	3.12 ±0.08	1.58 ±0.05
		3	1.56 ±0.05	2.85 ±0.10	2.45 ±0.06
		5	1.68 ±0.24	4.15 ±0.13	3.66 ±0.09
		10	1.42 ±0.10	4.15 ±0.25	4.02 ±0.35

Table 13: Miniaturized system: spatial resolution sampled on the Y axis in the transverse plane. The extended energy range $\Delta E\,(ext)$ is used.

opening	FOV	position [mm]	axial [mm]	radial [mm]	tangential [mm]
0 mm	centre	0	1.13 ±0.08	1.13 ±0.05	1.13 ±0.05
		1	1.15 ±0.11	1.08 ±0.09	1.21 ±0.07
		2	1.15 ±0.12	1.65 ±0.12	1.25 ±0.09
		3	1.14 ±0.11	2.25 ±0.26	1.32 ±0.10
		5	1.16 ±0.14	2.83 ±0.47	1.39 ±0.14
		10	1.10 ±0.15	8.47 ±0.44	2.15 ±0.38
	one-fourth	0	2.75 ±0.15	1.14 ±0.05	1.15 ±0.05
		1	2.78 ±0.18	1.06 ±0.07	1.21 ±0.06
		2	2.78 ±0.24	1.58 ±0.11	1.26 ±0.08
		3	2.81 ±0.22	2.20 ±0.26	1.29 ±0.09
		5	2.78 ±0.34	2.99 ±0.41	1.38 ±0.12
		10	0.87 ±0.98	9.25 ±0.51	1.99 ±0.22
20 mm	centre	0	1.19 ±0.03	2.27 ±0.05	1.63 ±0.05
		1	1.18 ±0.04	2.17 ±0.07	1.62 ±0.05
		2	1.19 ±0.04	2.04 ±0.07	1.49 ±0.05
		3	1.19 ±0.04	1.65 ±0.10	1.64 ±0.05
		5	1.30 ±0.06	2.95 ±0.12	1.40 ±0.07
		10	1.34 ±0.07	2.02 ±0.11	1.25 ±0.07
	one-fourth	0	1.86 ±0.08	4.06 ±0.21	1.69 ±0.05
		1	1.85 ±0.07	2.24 ±0.14	1.58 ±0.05
		2	1.78 ±0.06	2.09 ±0.04	1.51 ±0.04
		3	1.86 ±0.07	1.67 ±0.10	1.56 ±0.06
		5	2.06 ±0.21	3.11 ±0.08	1.43 ±0.06
		10	1.88 ±0.11	2.06 ±0.08	1.30 ±0.06
40 mm	centre	0	1.15 ±0.03	4.09 ±0.14	1.19 ±0.04
		1	1.14 ±0.04	2.66 ±0.10	1.25 ±0.04
		2	1.16 ±0.03	3.26 ±0.14	1.26 ±0.04
		3	1.14 ±0.04	2.93 ±0.33	1.31 ±0.04
		5	1.16 ±0.04	3.70 ±0.20	1.35 ±0.06
		10	1.18 ±0.06	2.23 ±0.29	1.17 ±0.06
	one-fourth	0	1.56 ±0.06	7.10 ±0.33	1.25 ±0.03
		1	1.52 ±0.08	5.97 ±0.32	1.26 ±0.04
		2	1.52 ±0.13	3.85 ±0.13	1.27 ±0.04
		3	1.65 ±0.04	3.12 ±0.10	1.32 ±0.04
		5	1.81 ±0.07	3.80 ±0.12	1.28 ±0.04
		10	1.63 ±0.08	2.16 ±0.14	1.19 ±0.05

FBP results for spatial resolution
Conceptual System

Table 14: Conceptual system: Spatial resolution sampled on the X axis in the transverse plane. The traditional energy range $\Delta E\,(trad)$ is used with FBP reconstruction.

opening	FOV	position [mm]	axial [mm]	radial [mm]	tangential [mm]
0 mm	centre	0	7.29±0.69	4.2±0.2	4.39±0.21
		5	8.88±2.95	4±0.32	4.41±0.65
		10	9.56±3.4	4.16±0.37	6.57±0.2
		15	9.08±3.25	4.83±0.83	4.19±0.35
		25	8.42±0.97	5.31±0.71	6.07±0.71
		35	7.56±1.22	6.41±0.82	5.14±0.73
	one-fourth	0	7.51±0.77	4.18±0.21	4.38±0.22
		5	9.62±2.5	4.04±0.32	4.61±0.7
		10	9.72±2.09	4.09±0.38	6.72±0.21
		15	9.61±2.33	4.67±0.81	4.33±0.41
		25	8.66±1.84	5.22±0.71	6.36±0.93
		35	7.43±0.96	6.36±0.83	5.37±0.61
20 mm	centre	0	8.21±1.13	19.34±6.91	4.48±0.24
		5	9.84±3.1	13.93±1.78	4.95±0.47
		10	9.72±3.2	22.78±2.79	6.63±0.26
		15	9.27±2.48	21.21±3.1	4.39±0.4
		25	10.12±2.45	19.65±8.74	6±0.72
		35	9.49±2.71	20.94±1.65	5.44±0.58
	one-fourth	0	8.3±1.5	19.55±4.45	4.45±0.25
		5	10.04±3	14.15±1.84	4.96±0.48
		10	9.72±2.34	22.63±2.74	6.82±0.28
		15	9.29±2.46	21.32±3.11	4.49±0.4
		25	9.78±2.56	19.72±9.38	6.16±0.88
		35	9.21±2.3	20.96±1.57	5.65±0.66
40 mm	centre	0	8.34±1.4	10.82±1.14	4.45±0.21
		5	10.58±2.52	25.23±3.5	4.92±0.33
		10	9.98±2.56	39.64±2.55	6.61±0.27
		15	8.71±2.03	18±6.27	4.53±0.42
		25	8.94±2.28	33.18±3.84	6.28±0.69
		35	9.74±2.22	32.62±3.71	5.79±0.58
	one-fourth	0	8.29±1.45	10.9±1.25	4.46±0.23
		5	10.26±2.56	25.32±3.6	4.95±0.34
		10	10.03±2.34	39.67±2.52	6.64±0.29
		15	8.77±1.75	19.29±9.91	4.74±0.47
		25	8.74±1.73	34.08±4.11	6.52±1
		35	9.32±2.24	32.67±4.28	5.97±0.75

Table 15: Conceptual system: Spatial resolution sampled on the Y axis in the transverse plane. The traditional energy range $\Delta E\,(trad)$ is used with FBP reconstruction.

opening	FOV	position	axial [mm]	radial [mm]	tangential [mm]
0 mm	centre	0	7.26±0.67	4.22±0.2	4.37±0.21
		5	9.62±4.02	6.34±0.28	4.33±0.4
		10	9.16±2.95	5.5±0.73	4.56±0.58
		15	8.99±2.93	4.87±0.69	4.48±0.55
		25	8.42±0.99	4.1±0.1	4.62±0.48
		35	7.44±1.22	7.25±0.85	4.98±0.73
	one-fourth	0	7.5±0.76	4.21±0.21	4.37±0.22
		5	9.96±3.32	6.35±0.29	4.26±0.41
		10	9.59±2.15	5.69±0.31	4.54±0.58
		15	9.58±2.14	4.84±0.67	4.44±0.57
		25	8.83±2.13	4.31±0.38	4.62±0.49
		35	7.32±0.84	7.55±1.13	4.92±0.72
20 mm	centre	0	8.2±1.16	18.72±7.6	4.45±0.23
		5	10.23±3.58	8.32±2.08	4.36±0.34
		10	11.21±3.8	13.98±1.52	4.6±0.53
		15	9.97±3.42	4.19±0.1	4.58±0.52
		25	8.69±2.7	4.8±0.72	4.72±0.67
		35	7.9±1.46	3.67±0.42	4.86±0.63
	one-fourth	0	8.34±1.47	19.04±8.49	4.45±0.24
		5	10.14±3	8.37±2.14	4.36±0.36
		10	10.55±2.95	14.04±1.51	4.53±0.53
		15	9.43±2.35	4.3±0.08	4.53±0.53
		25	8.82±2.03	4.83±0.56	4.71±0.66
		35	7.72±1.68	3.73±0.44	4.85±0.63
40 mm	centre	0	8.33±1.38	10.97±1.19	4.46±0.22
		5	10.3±2.82	9.55±1.45	4.43±0.33
		10	10.68±2.57	19.84±2.54	4.55±0.4
		15	9.35±2.4	16.36±1.81	4.62±0.42
		25	9.88±2.67	4.22±0.09	4.88±0.63
		35	8.19±1.53	4.9±0.43	4.89±0.59
	one-fourth	0	8.4±1.48	10.96±1.29	4.46±0.23
		5	9.67±2.39	9.57±1.56	4.43±0.34
		10	10.23±2.56	19.84±2.52	4.53±0.41
		15	8.83±1.67	16.74±8.68	4.59±0.42
		25	9.65±2.52	4.28±0.09	4.86±0.65
		35	8.1±1.55	4.94±0.45	4.79±0.59

Table 16: Conceptual system:: spatial resolution sampled on the X axis in the transverse plane. The extended energy range $\Delta E\,(ext)$ is used with FBP reconstruction.

opening	FOV	position [mm]	axial [mm]	radial [mm]	tangential [mm]
0 mm	centre	0	7.89 ±0.44	4.43 ±0.13	4.65 ±0.24
		5	10.06 ±2.01	4.31 ±0.18	4.67 ±0.37
		10	10.49 ±1.76	4.40 ±0.2	6.64 ±0.11
		15	9.68 ±1.65	5.06 ±0.37	4.51 ±0.24
		25	8.77 ±1.63	5.48 ±0.33	6.52 ±0.45
		35	7.78 ±0.55	6.50 ±0.43	5.38 ±0.34
	one-fourth	0	7.85 ±0.2	4.44 ±0.04	4.60 ±0.04
		5	10.11 ±0.39	4.35 ±0.13	4.74 ±0.24
		10	10.21 ±1.39	4.35 ±0.18	6.75 ±0.38
		15	9.81 ±1.31	4.97 ±0.21	4.66 ±0.11
		25	8.93 ±1.37	5.41 ±0.36	6.64 ±0.24
		35	7.53 ±0.97	6.47 ±0.33	5.54 ±0.45
20 mm	centre	0	9.19 ±1.37	19.28 ±3.1	4.81 ±0.22
		5	11.16 ±1.83	15.68 ±0.87	5.16 ±0.24
		10	10.82 ±1.68	22.00 ±1.05	6.71 ±0.15
		15	10.06 ±1.58	20.47 ±3.14	4.70 ±0.24
		25	10.78 ±1.47	18.43 ±2.32	6.45 ±0.44
		35	10.06 ±1.43	20.51 ±1.39	5.72 ±0.37
	one-fourth	0	9.03 ±1.02	19.27 ±3.49	4.77 ±0.23
		5	10.68 ±1.35	15.81 ±0.87	5.23 ±0.25
		10	10.38 ±1.24	21.99 ±1.06	6.84 ±0.16
		15	9.88 ±1.25	20.79 ±2.15	4.87 ±0.3
		25	10.47 ±1.22	18.50 ±3.37	6.55 ±0.47
		35	9.70 ±1.22	20.54 ±0.99	5.89 ±0.38
40 mm	centre	0	9.85 ±1.35	16.92 ±1.07	4.76 ±0.19
		5	11.37 ±1.38	26.05 ±2.56	5.08 ±0.19
		10	10.80 ±1.31	39.10 ±2.16	6.59 ±0.15
		15	9.58 ±1.28	18.85 ±4.1	4.89 ±0.25
		25	9.72 ±1.25	32.17 ±1.54	6.75 ±0.55
		35	10.39 ±1.25	32.31 ±1.12	5.94 ±0.39
	one-fourth	0	9.30 ±1.17	16.97 ±1.03	4.77 ±0.21
		5	10.69 ±1.36	26.31 ±2.55	5.10 ±0.2
		10	10.48 ±1.07	39.07 ±2.16	6.61 ±0.21
		15	9.40 ±1.11	21.16 ±8.23	5.05 ±0.26
		25	9.39 ±1.24	32.41 ±1.4	6.84 ±0.6
		35	10.02 ±1.1	32.30 ±1.08	6.12 ±0.4

Table 17: Conceptual system: Spatial resolution sampled on the Y axis in the transverse plane. The extended energy range $\Delta E\,(ext)$ is used with FBP reconstruction.

opening	FOV	position	axial [mm]	radial [mm]	tangential [mm]
0 mm	centre	0	7.86±0.43	4.45±0.13	4.64±0.24
		5	10.41±2.07	6.53±0.16	4.61±0.25
		10	10.1±1.63	5.69±0.16	4.88±0.35
		15	9.63±1.68	5.2±0.39	4.77±0.33
		25	8.81±1.48	4.3±0.25	4.89±0.33
		35	7.62±0.47	7.45±0.51	5.17±0.37
	one-fourth	0	7.85±0.39	4.46±0.13	4.63±0.24
		5	10.28±1.32	6.53±0.16	4.55±0.26
		10	10.05±1.3	5.75±0.16	4.79±0.35
		15	9.77±1.21	5.2±0.38	4.69±0.33
		25	9.04±1.08	4.7±0.28	4.87±0.33
		35	7.39±0.42	7.56±0.53	5.11±0.37
20 mm	centre	0	9.13±1.37	19.16±3.16	4.81±0.22
		5	11.3±1.8	9.8±1.15	4.69±0.26
		10	11.76±1.67	13.84±0.82	4.95±0.3
		15	10.77±1.7	4.2±0.06	4.87±0.28
		25	9.5±1.44	5.09±0.4	5.06±0.36
		35	8.18±0.68	3.81±0.26	5.08±0.34
	one-fourth	0	9.01±1	19.11±3.55	4.78±0.22
		5	10.66±1.36	9.87±1.15	4.67±0.27
		10	11.24±1.81	13.9±0.83	4.88±0.3
		15	10.1±1.47	4.25±0.06	4.81±0.28
		25	9.39±1.11	5.18±0.48	5.07±0.37
		35	8.03±0.91	4.19±0.53	5.04±0.34
40 mm	centre	0	9.71±1.34	16.94±1.04	4.78±0.2
		5	11.19±1.32	12.02±1.76	4.72±0.22
		10	11.46±1.4	19.24±2.38	4.86±0.24
		15	10.12±1.34	17.26±2.31	4.9±0.24
		25	10.36±1.46	4.23±0.06	5.2±0.36
		35	8.77±1.11	5.13±0.23	5.11±0.31
	one-fourth	0	9.36±1.11	16.97±1.04	4.77±0.21
		5	10.32±1.17	12.07±1.99	4.72±0.22
		10	10.64±1.12	19.28±2.54	4.83±0.25
		15	9.64±1.33	17.13±1.3	4.86±0.24
		25	9.97±1.24	4.27±0.06	5.16±0.36
		35	8.55±0.85	5.19±0.23	5.01±0.31

FBP results for spatial resolution
Miniaturized System

Table 18: Miniaturized system: Spatial resolution sampled on the X axis in the transverse plane. The traditional energy range $\Delta E\,(trad)$ is used with FBP reconstruction.

opening	FOV	position	axial [mm]	radial [mm]	tangential [mm]
0 mm	centre	0	4.68±0.65	1.75±0.05	1.81±0.09
		1	4.58±1.06	1.83±0.07	1.69±0.05
		2	5.19±0.73	1.62±0.08	1.86±0.12
		3	4.34±0.89	1.64±0.08	1.32±0.08
		5	4.06±0.8	2.03±0.19	6.76±0.56
		10	5.03±0.82	2.82±0.19	4.45±0.32
	one-fourth	0	4.34±0.52	1.86±0.05	1.7±0.12
		1	4.35±0.56	1.93±0.06	1.65±0.06
		2	4.4±0.47	1.83±0.07	1.78±0.16
		3	4.29±0.5	1.71±0.06	1.48±0.1
		5	4.18±0.55	1.98±0.1	6.57±1.15
		10	4.4±0.59	2.64±0.18	4.53±0.33
20 mm	centre	0	3.57±0.27	17.22±0.03	1.54±0.05
		1	3.67±0.35	17.2±0.09	1.62±0.03
		2	4.58±0.34	17.97±0.05	1.75±0.06
		3	4.38±0.38	19.43±0.07	1.44±0.04
		5	3.75±0.38	16.95±0.04	3.2±0.16
		10	3.94±0.34	16.89±0.19	3.98±0.18
	one-fourth	0	3.25±0.26	16.93±0.04	1.48±0.04
		1	3.04±0.27	16.92±0.09	1.58±0.03
		2	3.91±0.29	17.85±0.06	1.83±0.06
		3	3.81±0.34	19.45±0.06	1.43±0.04
		5	3.71±0.35	17.08±0.04	3.33±0.22
		10	3.39±0.33	16.94±0.18	4.25±0.2
40 mm	centre	0	3.05±0.49	24.63±1.68	1.4±0.07
		1	3.06±0.46	24.32±0.22	1.53±0.07
		2	3.48±0.57	25.38±0.14	1.68±0.1
		3	3.84±0.67	25.93±0.14	1.51±0.1
		5	3.88±0.78	25.68±0.19	3.77±0.59
		10	3.61±0.7	24.27±0.32	3.57±0.38
	one-fourth	0	3.55±0.5	25.07±0.26	1.41±0.1
		1	3.17±0.43	23.66±0.35	1.65±0.11
		2	3.54±0.47	24.31±0.25	1.86±0.18
		3	3.42±0.44	24.89±0.15	1.49±0.13
		5	3.27±0.46	24.32±0.17	3.62±0.54
		10	3.48±0.65	24.09±0.35	4.02±0.42

Table 19: Miniaturized system: Spatial resolution sampled on the Y axis in the transverse plane. The traditional energy range $\Delta E\,(trad)$ is used with FBP reconstruction.

opening	FOV	position	axial [mm]	radial [mm]	tangential [mm]
0 mm	centre	0	4.63±0.65	1.63±0.05	1.78±0.09
		1	4.69±0.69	2.68±0.08	1.73±0.1
		2	4.71±0.91	2.36±0.15	1.64±0.1
		3	4.86±0.7	3.31±0.2	1.7±0.1
		5	4.79±0.77	3±0.26	1.6±0.1
		10	4.69±0.93	2.73±0.3	1.92±0.15
	one-fourth	0	4.44±0.58	1.67±0.05	1.68±0.12
		1	4.71±0.54	2.43±0.07	1.59±0.11
		2	5.32±0.77	2.45±0.12	1.58±0.12
		3	4.63±0.79	3.56±0.21	1.69±0.12
		5	4.29±0.49	3.4±0.26	1.66±0.13
		10	3.72±0.56	3.22±0.41	1.9±0.16
20 mm	centre	0	3.23±0.28	5.8±0.03	1.49±0.06
		1	3.4±0.27	4.33±0.04	1.51±0.07
		2	3.98±0.65	4±0.03	1.42±0.06
		3	3.8±0.44	7.02±0.05	1.51±0.08
		5	3.31±0.35	2.4±0.1	1.56±0.08
		10	3.38±0.44	15.75±0.05	1.62±0.1
	one-fourth	0	3.4±0.28	5.83±0.03	1.38±0.06
		1	3.67±0.4	4.17±0.04	1.48±0.07
		2	3.64±0.37	4.26±0.03	1.5±0.08
		3	3.72±0.35	7.23±0.04	1.46±0.08
		5	3.22±0.27	2.59±0.11	1.57±0.11
		10	2.91±0.21	17.33±0.06	1.71±0.12
40 mm	centre	0	2.6±0.25	6.45±0.09	1.4±0.05
		1	3.04±0.24	6.3±0.11	1.33±0.05
		2	2.94±0.28	10.19±0.08	1.34±0.05
		3	2.85±0.26	8.35±0.08	1.45±0.06
		5	2.75±0.27	26.82±0.36	1.38±0.06
		10	3.67±0.47	25.51±0.03	1.48±0.09
	one-fourth	0	2.86±0.19	9.95±0.08	1.34±0.05
		1	3.02±0.2	8.12±0.09	1.33±0.05
		2	3.18±0.2	10.28±0.06	1.33±0.06
		3	3.06±0.19	8.77±0.04	1.44±0.07
		5	2.74±0.21	25.75±0.19	1.43±0.08
		10	3.11±0.33	25.64±0.03	1.53±0.13

Table 20: Miniaturized system: Spatial resolution sampled on the X axis in the transverse plane. The extended energy range $\Delta E\,(ext)$ is used with FBP reconstruction.

opening	FOV	position	axial [mm]	radial [mm]	tangential [mm]
0 mm	centre	0	5.11±0.2	1.94±0.05	1.89±0.04
		1	5.05±0.21	1.9±0.07	1.73±0.02
		2	5.39±0.24	1.84±0.09	1.9±0.04
		3	5.04±0.26	1.85±0.09	1.49±0.06
		5	4.54±0.22	2.09±0.17	3.89±0.18
		10	4.78±0.23	2.6±0.3	4.3±0.12
	one-fourth	0	4.31±0.2	1.95±0.05	1.76±0.04
		1	4.39±0.19	1.91±0.07	1.72±0.02
		2	4.66±0.18	1.85±0.07	1.89±0.05
		3	4.54±0.19	1.84±0.07	1.75±0.09
		5	3.91±0.18	2.08±0.13	4.9±0.35
		10	3.87±0.18	2.4±0.26	4.23±0.13
20 mm	centre	0	4.67±0.11	17.24±0.03	1.71±0.02
		1	4.67±0.11	17.18±0.06	1.72±0.01
		2	5.26±0.12	17.67±0.06	1.89±0.02
		3	5.03±0.13	18.43±0.09	1.53±0.02
		5	4.71±0.15	16.12±0.07	3.41±0.06
		10	4.41±0.14	16.27±0.24	4.12±0.06
	one-fourth	0	4.16±0.11	16.99±0.12	1.67±0.02
		1	4.07±0.11	16.94±0.06	1.69±0.01
		2	4.63±0.13	17.46±0.07	1.89±0.02
		3	4.46±0.13	18.38±0.08	1.56±0.06
		5	4.27±0.14	15.8±0.06	3.5±0.07
		10	4.1±0.13	16.18±0.25	4.24±0.07
40 mm	centre	0	3.87±0.21	24.39±0.15	1.55±0.05
		1	3.75±0.21	24.14±0.1	1.64±0.03
		2	4.07±0.22	24.48±0.09	1.76±0.05
		3	4.25±0.23	24.91±0.09	1.89±0.13
		5	4.33±0.26	23.96±0.18	3.6±0.17
		10	3.88±0.28	22.81±0.58	3.78±0.11
	one-fourth	0	3.52±0.17	23.85±0.3	1.51±0.05
		1	3.34±0.16	21.26±0.26	1.66±0.04
		2	3.54±0.17	23.97±0.15	1.82±0.06
		3	3.56±0.22	24.22±0.09	1.56±0.24
		5	3.53±0.21	23.29±0.22	3.56±0.22
		10	3.3±0.17	22.15±0.2	3.63±0.13

Table 21: Miniaturized system: Spatial resolution sampled on the Y axis in the transverse plane. The extended energy range $\Delta E\,(ext)$ is used with FBP reconstruction.

opening	FOV	position	axial [mm]	radial [mm]	tangential [mm]
0 mm	centre	0	4.81±0.2	1.89±0.05	1.86±0.03
		1	4.82±0.22	2.67±0.08	1.77±0.04
		2	5.32±0.26	2.56±0.12	1.73±0.04
		3	5.12±0.23	3.59±0.25	1.83±0.05
		5	4.81±0.25	3.21±0.4	1.71±0.05
		10	4.35±0.25	2.63±0.62	1.97±0.04
	one-fourth	0	4.34±0.2	1.87±0.05	1.72±0.04
		1	4.63±0.32	2.45±0.08	1.71±0.04
		2	4.71±0.32	2.45±0.12	1.66±0.04
		3	4.47±0.19	3.65±0.27	1.71±0.05
		5	4.15±0.17	3.22±0.42	1.67±0.05
		10	3.61±0.18	2.98±0.63	1.92±0.03
20 mm	centre	0	3.87±0.16	6.14±0.03	1.63±0.03
		1	4.16±0.17	5.98±0.03	1.66±0.03
		2	4.48±0.21	5.18±0.04	1.61±0.03
		3	4.25±0.17	7.39±0.06	1.65±0.04
		5	3.88±0.15	2.45±0.1	1.68±0.04
		10	3.91±0.17	11.95±0.08	1.7±0.04
	one-fourth	0	3.57±0.14	5.97±0.03	1.57±0.04
		1	3.73±0.14	5.69±0.03	1.61±0.04
		2	3.92±0.14	5.17±0.03	1.59±0.04
		3	3.77±0.13	7.51±0.05	1.62±0.04
		5	3.38±0.09	2.52±0.11	1.64±0.04
		10	3.25±0.16	12.83±0.09	1.73±0.03
40 mm	centre	0	3.22±0.09	10.61±0.06	1.52±0.02
		1	3.58±0.12	8.49±0.03	1.47±0.02
		2	3.53±0.13	13.69±0.07	1.45±0.04
		3	3.45±0.1	8.79±0.05	1.58±0.03
		5	3.29±0.11	17.23±0.21	1.51±0.05
		10	3.59±0.19	23.15±0.17	1.62±0.02
	one-fourth	0	3.03±0.07	10.66±0.06	1.48±0.02
		1	3.22±0.08	8.31±0.03	1.45±0.02
		2	3.27±0.08	11.86±0.05	1.42±0.04
		3	3.22±0.07	8.85±0.04	1.57±0.04
		5	2.96±0.08	21.81±0.16	1.54±0.05
		10	3.1±0.11	22.33±0.28	1.6±0.05
